# Correlates of co-occurring physical child punishment and physical intimate partner violence in Colombia, Mexico and Peru

**DOI:** 10.1186/s12889-022-14453-6

**Published:** 2022-11-28

**Authors:** Sarah Bott, Ana P. Ruiz-Celis, Jennifer Adams Mendoza, Alessandra Guedes

**Affiliations:** 1 Independent Consultant, Los Angeles, CA USA; 2 Independent Consultant, Mexico City, Mexico; 3 Independent Consultant, Knoxville, TN USA; 4 UNICEF, Office of Research—Innocenti, Piazza SS Annunziata 12, 50121 Florence, Italy

**Keywords:** Child maltreatment, Intimate partner violence, Epidemiology, Latin America and the Caribbean

## Abstract

**Background:**

Violent discipline of children and intimate partner violence (IPV) against women are global public health and human rights problems. To address calls for more evidence on intersections, this study aimed to expand knowledge about correlates of physical child punishment, physical IPV against women and their co-occurrence (both) in the same household.

**Methods:**

Using national, population-based survey datasets from Colombia, Mexico and Peru, multinomial logistic regressions examined correlates of three mutually exclusive patterns of violence in the household: physical child punishment (only), physical IPV ever (only) and co-occurrence (both), each compared with no violence, after adjusting for other factors. Logistic regression was used to analyse odds ratios of physical child punishment in households affected by IPV past year and before past year compared with never, after adjusting for other factors.

**Results:**

In all countries, adjusted odds ratios (aOR) of co-occurrence were significantly higher among women with lower education, more than one child, a child aged 2–5, a partner who tried to socially isolate her, and a history of childhood violence (caregiver violence and/or IPV exposure). They were significantly lower among women who reported collaborative partnerships (joint decision-making and/or shared chores). Co-occurrence was also significantly correlated with a history of child marriage/early motherhood in Colombia and Mexico, partner’s excess drinking in Mexico and Peru, agreement that physical child punishment was necessary in Peru and partner’s history of childhood violence in Colombia and Mexico. Evidence of shared risk factors was strongest for social isolation and caregiver histories of childhood violence and of shared protective factors for collaborative partnership dynamics. In all countries, associations between physical child punishment and physical IPV remained significant after adjusting for other factors, suggesting that correlations could not be explained by shared risk factors alone.

**Conclusions:**

These findings are consistent with several theories relevant for violence prevention: 1) more collaborative, gender equitable partnerships may protect both children and women from violence; 2) violence between intimate partners may ‘spill over’ into violence against children (as correlations could not be explained by shared risk factors alone); and 3) there appears to be strong evidence of intergenerational transmission of violence.

**Supplementary Information:**

The online version contains supplementary material available at 10.1186/s12889-022-14453-6.

## Background

The international community recognizes violent discipline of children and intimate partner violence (IPV) against women as global public health and human rights problems [1, 2]. As part of 2030 Sustainable Development Goal (SDG) commitments [3], all United Nations (UN) Member States agreed to work towards eliminating these forms of violence and measure progress through national data collection [4]. As of January 2021, Colombia and Peru had banned corporal punishment of children in all settings including the home, and Mexico had taken legislative steps towards full prohibition [5].

Violent discipline, defined by the UN as physical punishment and/or verbal aggression by caregivers, is the most common form of violence against children globally [6, 7]. Within Latin America and the Caribbean (LAC), an estimated 50–60% of 2–3 year old children are regularly physically punished, declining to 30–40% by age 14 [8]. Meanwhile, IPV is the most widespread form of violence against women [9], with national estimates of physical IPV (ever) against ever partnered women in LAC ranging from 7% in Uruguay to 52% in Bolivia [10].

Both violent discipline and exposure to IPV have negative consequences for children [11–14]. Metanalyses have found associations between physical child punishment and cognitive, behavioural, mental health and emotional problems throughout the lifespan, even when physical punishment does not meet legal definitions of child abuse [13, 14]. Similarly, exposure to IPV has been linked to children’s emotional impairment and mental health disorders [15], malnutrition and stunting [16], aggression towards peers and siblings [17] and negative perinatal health outcomes [18].

Preliminary evidence suggests that*** co-occurrence*** —defined in this article as caregiver violence against children and IPV against women in the same household— may compound negative effects of either violent discipline or exposure to IPV alone [19–21]. For example, a study from Uganda found that children who witnessed IPV and experienced violence themselves had about twice the odds of mental health difficulties as children who experienced violence but did not witness IPV [22].

Based on growing evidence of intersections between violence against children and violence against women [23], some question the feasibility of achieving the SDGs without a better understanding of the complex inter-relationships among factors associated with violence against children and violence against women [24]. A 2020 review identified 132 studies on correlates of co-occurring IPV and child maltreatment in the same household [21]; however, studies used enormously diverse research methods and definitions of violence, making findings difficult to compare. Moreover, the vast majority came from high-income settings. In fact, a 2022 systematic review found only three studies from low- and middle-income countries (LMICs) that explored risk factors for co-occurrence [25]. Studies from high-income countries may have limited generalizability to LMICs, both because drivers of violence may differ [26], and because studies from high-income settings often (though not always) focus on at-risk populations and/or severe cases of child or partner abuse reported to authorities, rather than common forms of violent discipline captured by population-based surveys [21, 27].

One barrier to understanding co-occurrence in LMICs is that most high quality, national data on violent discipline come from UNICEF-supported Multiple Indicator Cluster Surveys (MICS) [28], which do not usually measure IPV prevalence. As a result, the growing number of multi-country analyses of MICS data have not included child exposure to IPV as a potential risk factor in research on violent discipline [29–36]. Conversely, most national surveys that measure IPV against women do not measure child discipline, with some exceptions, including the surveys in this study.

Nonetheless, research on co-occurrence in LMICs is growing. Some studies examine IPV against mothers as a correlate of violent discipline [37–40]. Others examine correlates of co-occurrence itself [25, 26, 41, 42]. This research suggests several key findings and knowledge gaps. First, many studies find that children in households affected by IPV are more likely than other children to experience violent discipline [37–40, 43–45]. What pathways explain these associations are not entirely understood, however. Some researchers suggest abused women may discipline children more harshly due to depression, stress, anxiety, trauma or maternal alcohol abuse [26, 42, 46–48]. Others theorize that men who abuse their partners may be more likely to abuse their children [19]. Some researchers propose a ‘spillover hypothesis’, whereby violent norms and behaviours in intimate partnerships spill over into caregiver-child relationships due to stress, social learning or attitudes normalizing violence [21, 27, 46, 49].

Others suggest that associations reflect shared risk factors that independently increase the risk of each form of violence [19, 23, 26]. Proposed shared risk factors include legal frameworks that fail to protect the rights of women or children [33, 50–53], high levels of crime, armed violence or socio-economic disadvantages in communities [42, 54–58], social norms supportive of violence or gender inequality within society [35, 50, 51] or households [23, 29, 31, 36, 59–62], patriarchal family structures [46, 63]; men’s harmful use of alcohol [23, 64–66], caregiver histories of violence in childhood [23, 37, 40, 55, 58, 64, 67–74], and social isolation, including during COVID-19 lockdowns [75–78].

Meanwhile, some correlates commonly explored in research on violence against children, including caregiver agreement that physical punishment is necessary for child rearing [29, 36, 59, 61], are rarely explored in IPV research. Conversely, some documented correlates of IPV such as partners’ controlling behaviour (e.g. preventing women from seeing friends and family) [69, 70, 79] and women’s histories of child marriage [80] have not been widely explored in research on violent discipline of children.

Another complexity is that gender inequality is widely theorized to be a driver of both violence against children and violence against women in LMICs by researchers [23, 46, 63, 81] and by international frameworks for violence prevention [82, 83], but measuring this domain poses challenges. For example, researchers sometimes use female participation in household decision-making as a proxy for women’s empowerment [84], but others question its validity [85]. Moreover, some studies have found higher levels of IPV among women who make household decisions alone than among women who report joint decision-making with their partner, suggesting that it may be collaborative rather than any female decision-making that is most protective for IPV [63, 65, 79, 86, 87]. In addition, researchers often find complex, nonlinear relationships between levels of violence and proxies for women’s empowerment such as education, wealth or earning power [63, 70, 81, 88].

To explore co-occurrence and possible shared risk factors, studies from Brazil [42] and Uganda [26] analysed correlates of three mutually exclusive patterns of violence in the household: caregiver violence against children (only), IPV against women (only), and both forms (co-occurrence). Using a population-based birth cohort of 3,533 mothers with four-year-old children, the Brazilian study found associations between co-occurrence and neighbourhood violence, absence of biological father, antisocial paternal behaviour, mother-partner relationships characterised by high levels of criticism, maternal depression and younger maternal age. Among 535 adolescent-caregiver dyads, the Ugandan study found associations between co-occurrence and women’s lower socioeconomic status, lower education, higher mental distress and frequent alcohol use, male caregiver support for physical child punishment and lower emotional attachment between partners. These studies focused on different child cohorts (four-year-olds versus adolescents) and used starkly different definitions of violence, making them hard to compare. Nonetheless, commonalities in their approach suggests a promising way to begin disentangling links between different forms of violence in the household.

### Aims and research questions

Building on these studies from Brazil and Uganda, this article presents a secondary analysis of three national, population-based household surveys from Colombia, Mexico and Peru. This study aimed to expand what is known about correlates of IPV against women and physical punishment of children by addressing the following research questions:

- 1. Which risk and protective factors were associated with co-occurring physical IPV against women and physical punishment of children in the same household?
- 2. Which correlates were ‘shared’ across three patterns of physical violence in the household (physical child punishment only, physical IPV only, and co-occurrence), each compared with no violence?
- 3. Is physical IPV against women in the household a significant risk factor for physical child punishment, after controlling for other factors?

Henceforth this article refers to women and girls aged 15–49 as ‘women’ for simplicity, while acknowledging that the Convention on the Rights of the Child considers girls below age 18 to be children not adults, even if they have already married or borne children [1].

## Methods

### Survey characteristics

This study involved a secondary analysis of three national, open access, population-based household surveys: the 2015 Colombia Demographic and Health Survey (DHS) [89], the 2016 Mexico *Encuesta Nacional Sobre la Dinámica de las Relaciones en los Hogares* (ENDIREH) [90] and the 2018 Peru DHS [91]. These surveys were identified through a systematic search, described elsewhere [43] as the only surveys from LAC that gathered national co-occurrence data among women of reproductive age between 2014 and 2019. All three were implemented through collaborations between national governments and civil society. The 2016 Mexico ENDIREH was dedicated specifically to violence against women. The 2015 Colombia DHS and 2018 Peru DHS were health surveys that included a domestic violence module. Henceforth each survey is referred to by country name.

### Sample design

All three surveys used multi-stage, probability samples. Primary sampling units (PSUs) were selected from master sampling frames, usually a census. Households were randomly selected within each PSU. Full surveys included women aged 13–49 years in Colombia, 15+ years (with no age limit) in Mexico and 15–49 years in Peru. Mexico and Peru randomly selected one woman per household for questions about violence; Colombia included all ever-partnered, age-eligible women. Women were interviewed face-to-face, usually in or around the woman’s residence. All surveys had response rates above 85% and included weights for producing nationally representative estimates for women, children and households. Original survey reports provide more detail about sample design [92–94].

To maximize comparability and focus on households at greatest risk of co-occurrence, this analysis limited study subsamples to women: a) asked about both IPV and child discipline in the current household; b) currently married or living with a partner as if married; c) aged 15–49; d) living with a daughter or son aged 1–14. After standardization, study subsamples sizes were 13,518 in Colombia, 38,097 in Mexico and 17,156 in Peru (Table 1).

**Table 1:** Denominator calculations

	**Colombia 2015 DHS**	**Mexico 2016 ENDIREH**	**Peru 2018 DHS**
**Original full survey sample**	38,087	111,256	38,777
**Original child discipline sample**	21,537	90,068	22,412
*Original eligibility criteria for child discipline questions*	*Ever partnered and ever had a live birth*^a^	*Did not select response option ‘no children’*	*Selected for violence module and living with 1*+ *daughter or son*
**Reason excluded:**			
No child discipline data^b^	(16,550)	(21,188)	(16,365)
Never partnered^c^	0	(3,986)	(957)
Not age 15–49	(4)	(31,061)	0
Not a regular household member	0	0	(184)
Not living with a daughter or son aged 1–14	(4,463)	(11,926)	(1,770)
Previously but not currently partnered	(3,552)	(4,998)	(2,345)
**Study subsample, this analysis**	13,518	38,097	17,156

All numbers are unweighted

^a^These criteria matched the Colombia dataset but were different than filter instructions written on the questionnaire (living with 1+ biological or stepchild)

^b^Women without child discipline data included those who were not eligible, not selected, refused participation or reported “no children”

^c^Defined as never married or cohabited

### Safety and ethics

All three surveys were developed with participation of civil society organizations and advocates working on violence against women. All surveys adhered to World Health Organization ethical and safety recommendations for IPV research such as informed consent, privacy, confidentiality, female interviewers and interviewer training on violence against women [95]. However, Colombia asked all eligible women in the household about IPV not just one, posing a risk to confidentiality, and the informed consent process in Mexico did not emphasize women’s right to refuse any question. Published reports and manuals did not say whether interviewers made referrals to children’s services when respondents disclosed violence against children.

### Measures of physical punishment

Surveys measured physical child punishment in diverse ways (Table 2). Colombia and Peru asked open-ended questions about ***who*** punishes (“*castiga*”) the children before asking ***how*** and recorded answers with pre-coded categories. Mexico asked respondents whether they or their husbands/partners hit their children when they became ‘angry’ or ‘desperate’ (“*se enoja o desespera*”). All three surveys asked about punishment of daughters and sons generally, with no specific timeframe.

**Table 2:** Survey items used to measure physical punishment of children

**Survey items**^a^ (Translated by authors)
**Colombia**
Who punishes your sons (daughters) (or your step or adopted sons (daughters)) in the household? Anyone else?
father/stepfather; mother/respondent/ stepmother; other; no one (not punished)
if father/stepfather: How does your partner punish your (step, adopted) sons (daughters)?
if respondent: How do you punish your (step, adopted) sons (daughters)?
if other: How does that person punish your (step, adopted) sons (daughters)?
**coded acts of physical punishment**^b^: spank/smack; push; beat with objects
**Mexico**
When your husband or partner becomes angry or desperate with his/your daughters and sons, does he hit them sometimes? frequently? does not hit them? no sons/daughters?
When you become angry or desperate with your daughters and sons, do you hit them sometimes? frequently? do not hit them? no sons/daughters?
**coded acts of physical punishment**: sometimes hits; frequently hits
**Peru**
Who reprimands or punishes your daughters or sons in the household? Anyone else?
biological father; biological mother; other; no one (not punished)
if father: How does your husband/partner punish your daughter(s) or son(s)?
if mother: How do you punish your daughter(s) or son(s)?
if other: How does that person punish your daughter(s) or son(s)?
**coded acts of physical punishment**^b^: spank/smack; beat or ‘physical punishment’

^a^Words in small caps font were coded but not read to respondents

^b^Colombia and Peru also coded non-violent acts of discipline not listed in this table

For the secondary analysis,*** physical punishment*** was defined as any act of physical child discipline measured, except ‘pouring water’ (“*echándoles agua”*) in Peru, since it did not clearly meet the UN definition of ‘corporal punishment’ [96]. Otherwise eligible women in Colombia and Peru who reported that ‘no one’ punished the children were retained in denominators and coded as ‘no physical punishment’, in keeping with the MICS but in contrast to the 2018 Peru DHS report [93].

### Measures of physical IPV

This analysis was limited to physical IPV because surveys measured physical but not sexual or emotional IPV in highly comparable ways. All surveys used a modified conflict tactics scale [97], asking whether the woman’s current husband/partner had carried out specific acts (e.g. slapped, punched, etc.) ever and in the past 12 months (Table 3). The secondary analysis created two composite variables: a) physical IPV ever (dichotomous), defined as 1 = 1+ act by the current husband/partner ever and 0 = no acts by the current husband/partner ever (reference category); and b) physical IPV by timeframe (trichotomous) with three mutually exclusive timeframes: 1 = past year, 2 = before but not during the past year, and 0 = never (reference category). Women who completed the domestic violence module (or equivalent in Mexico) but did not respond to individual IPV questions were retained in denominators and classified as ‘no’ for that act, in keeping with the DHS.

**Table 3:** Operational definitions of physical intimate partner violence (IPV) in this analysis

**Survey**	**Acts of physical IPV included in the operational definition**
	Her current husband or partner (ever or in the past year):
Colombia	shoved or shook her; hit her with his hand; hit her with an object; kicked or dragged her; threatened her or attacked her with a knife, firearm, or other weapon; tried to strangle or burn her
Mexico	shoved her or pulled her hair; slapped or smacked her; tied her up; kicked her; threw an object at her; hit her with a fist or object; tried to choke or strangle her; threatened her with a knife or firearm or with burning; attacked her with a knife or razor; or shot her with a firearm
Peru	shoved, shook or threw something at her; slapped her or twisted her arm; hit her with his fist or something that could hurt her; kicked or dragged her; tried to strangle or burn her; attacked or threatened her with a knife, firearm, or other weapon

### Patterns of violence in the household

The key dependent variable in this analysis was ***pattern of physical violence in the household***, with four mutually exclusive categories: 1 = physical child punishment only (no physical IPV ever); 2 = co-occurrence (physical child punishment and physical IPV ever); 3 = physical IPV ever only (no physical child punishment); and 0 (reference category) = no violence (neither physical IPV ever nor physical child punishment). A modified version limited to severe physical child punishment (beating with objects) was created for Colombia to explore how findings compared with previously published analyses of that dataset [55].

### Potential correlates

Potential correlates were selected based on available data, previous research, variables found to be significant in bivariate analyses, testing for collinearity and covariance using variance inflation factor (VIF) analysis without design effects, and goodness of fit testing, with stepwise selection and elimination. Due to a lack of available tests for multinomial regression, goodness of fit testing was carried out using logistic regression after converting co-occurrence into a dichotomous variable (1 = co-occurring physical child punishment and physical IPV ever; 0 = neither of these).

Most sociodemographic variables were highly comparable across surveys, but other potential correlates were not (Table 4). All variables were constructed as dichotomous or categorical, not continuous, because many had a nonlinear relationship with violence. Missing responses were rare except for married/mother age < 18 (Supplemental Table A). For dichotomous variables, women missing data were retained in denominators and coded as not having that characteristic. Only one woman (from Mexico) was missing data for a categorical variable (education); she was excluded from regressions analyses.

**Table 4:** Operational definitions of potential correlates

**Variable**	**Operational definition**
**Women’s Age**	Age group (15–29 years; 30–39 years; and 40–49 years)
**Women’s education**	Highest level attended (primary or none; ≤ 3 years of lower secondary; > 3 years of upper secondary; any post-secondary)
**Household wealth**	Wealth terciles (poorest; middle; richest) generated by principal component analysis
**Residence**	Based on National Statistics Office (NSO) definitions
Colombia and Peru	(1 = urban; 0 = rural)
Mexico	(1 = urban; 2 = semi-urban; 0 = rural)
**Indigenous ethnicity**	(1 = indigenous; 0 = not indigenous)
Colombia	Indigenous by culture, community or physical characteristics, per household questionnaire
Mexico	Speaks indigenous language, per household questionnaire
Peru	Maternal tongue was indigenous, per women’s questionnaire
**Married/mother age < 18**	(1 = married, cohabited and/or gave birth at age < 18; 0 = did not marry, cohabit or give birth at age < 18)
**Number of children**	(1 = more than one daughter/son aged 1–14 living in the household; 0 = only one)
**Age of youngest child**	Age of youngest age-eligible (1–14) daughter/son living in the household in years (1; 2–5; 6–9; 10–14)
**Husband/partner tries to socially isolate her** (1 = yes; 0 = no)	
Colombia	He has prevented her from meeting friends or tried to limit her contact with her family in the past year
Mexico	He gets angry if she visits or goes out with friends or family
Peru	He prevents her from visiting friends or tries to limit her visits or contact with her family
**Husband/partner drinks to excess** (1 = yes; 0 = no)	
Colombia	*Not measured*
Mexico	She gets mad at her husband/partner because he drinks alcohol or takes drugs
Peru	Her partner gets drunk sometimes or frequently
**Joint decisions about money** (1 = yes; 0 = no)	
Colombia, Peru	She and her husband/partner both have a say in decisions about large purchases ***and*** daily necessities
Mexico	She and her husband/partner each have an ***equal*** say in deciding how to spend and save money
**She and her husband/partner share responsibility for household chores** (1 = yes; 0 = no)	
Colombia	They equally divide responsibility for cleaning the house, cooking ***or*** washing
Mexico	They are ***both*** responsible for domestic chores such as cleaning, cooking, ironing and washing
Peru	*Not measured*
**She agrees wife-beating is justified for 1+ reason** (1 = yes; 0 = no, don’t know or unsure all reasons)	
Colombia and Peru	Agrees with 1+ of the following reasons: 1. she leaves without telling him; 2. she neglects the children; 3. she argues with him; 4. she refuses sex; 5. she burns the food
Mexico	*Not measured*
**She believes physical punishment is necessary** (1 = sometimes or frequently; 0 = no or never)	
Colombia, Mexico	*Not measured*
Peru	She believes physical punishment is necessary for raising a child
**Violence in respondent’s childhood** (1 = caregiver violence only; 2 = co-occurrence; 3 = IPV only; 0 = none)	
***Caregiver violence against respondent in childhood*** (1 = yes; 0 = no)	
Colombia	Her parents or stepparents punished her by spanking/smacking, pushing or beating with objects
Mexico	People she lived with before age 15 hit her sometimes or often
Peru	Her parents punished her by spanking/smacking, beating or burning
***Respondent exposed to physical IPV in childhood*** (1 = yes; 0 = no or doesn’t know)	
Colombia	Her father beat her mother
Mexico	Adults in her childhood home hit or beat each other sometimes or frequently
Peru	Her father ever hit her mother
**Violence in husband/partner’s childhood** (1 = caregiver violence only; 2 = co-occurrence; 3 = IPV only; 0 = none)	
***Caregiver violence against husband/partner in childhood*** (1 = yes; 0 = no or doesn’t know)	
Colombia	He was mistreated/abused ("*maltratada*") by his parents or stepparents as a child
Mexico	He was hit or insulted in his childhood household sometimes or often before age 15
Peru	*Not measured*
***Husband/partner exposed to physical IPV in childhood*** (1 = yes; 0 = no or doesn’t know)	
Colombia	His father beat his mother
Mexico	His mother was hit/beaten by her husband before age 15
Peru	*Not measured*

*IPV* intimate partner violence

#### Sociodemographic variables

Women’s age was categorical, grouped as: 15–29, 30–39 and 40–49 years. Education was defined by highest level reached (not necessarily completed) categorized as primary or less, lower secondary, upper secondary and any post-secondary. Residence was based on national statistics office definitions: urban/rural in Colombia and Peru, and urban, semi-urban and rural in Mexico. The reduced model for Mexico combined urban and semi-urban for a more comparable analysis.

Household wealth was categorized as poorest, middle and richest terciles of wealth index scores. Wealth scores were calculated using principal components analysis of household assets and other characteristics. These were pre-coded by original research teams in Colombia and Peru. In Mexico, they were produced by authors (ARC, JAM) using SPSS (Version 26) following DHS methodology [98].

Indigenous ethnicity was based on culture, community or physical characteristics per the household questionnaire in Colombia; whether women spoke an indigenous language per the household questionnaire in Mexico; and indigenous maternal tongue per the woman’s questionnaire in Peru. Mexico and Peru also measured indigenous ethnicity based on culture or ancestry, used for sensitivity testing.

#### Reproductive histories

Married or mother < age 18 was based on whether the respondent gave birth and/or married/cohabited with a husband/partner before age 18. Child marriage/early motherhood was chosen instead of child marriage alone because a substantial proportion of girls in LAC give birth before marriage/cohabitation [99], and child marriage and early motherhood covaried, making it problematic to include them both in the same model as separate variables. In keeping with the DHS, missing data for this variable were imputed if possible, using variables such as respondent’s age at the time of the interview and age of the first born child.

More than one age-eligible child (1 = two or more; 0 = only one) was based on the number of daughters or sons aged 1–14 living in the household at the time of the interview.

Age of youngest child was defined as the age of the youngest daughter or son aged 1–14 living in the household drawn from the birth history in Colombia and Peru, and the household register in Mexico. Because physical child punishment had a nonlinear relationship with age of youngest child, a categorical variable was constructed with four mutually exclusive categories: age 1 (reference category); age 2–5; age 6–9; and age 10–14.

#### Partnership dynamics

Four dichotomous variables were constructed to measure partnership dynamics. All three surveys measured: a) partner tries to socially isolate her; and b) she and her partner make joint decisions about household finances. In addition, Mexico and Peru measured her partner’s excess drinking (Colombia did not). Colombia and Mexico also measured whether the respondent and her partner shared responsibility for key household chores (Peru did not). Surveys measured these variables in diverse ways (Table 4).

#### Social norms

Two dichotomous variables were constructed for social norms. Colombia and Peru measured women’s agreement that wife-beating was justified for at least one of five reasons. Peru also measured whether women agreed that physical punishment was necessary for child rearing, the sole variable measured by just one survey, included because of its importance in research on violent discipline [29, 36, 59, 61].

#### Histories of violence in childhood

Two variables for caregivers’ exposure to violence in childhood were constructed, one for respondents and one for partners, each with four mutually exclusive categories: 1 = caregiver violence only (no IPV exposure); 2 = co-occurrence (both); 3 = IPV exposure only (no caregiver violence); and 0 = no violence (reference category). These domains were measured in diverse ways, and Peru did not measure violence in the partner’s childhood. Violence in the respondent’s childhood and violence in her partner’s childhood covaried however, so a combined variable was constructed for sensitivity testing with 0 = no violence in either of their childhoods; 1 = co-occurrence in both of their childhoods; and 2 = all other patterns of childhood violence.

### Analysis

All analyses were done with Stata 16 (StataCorp LP, Texas) except for household wealth scores in Mexico, as previously noted. Analyses applied weights from domestic violence modules (Colombia and Peru) or the women’s dataset (Mexico), normalized to equalize weighted and unweighted numbers of women in each study subsample.

Percent distributions of each pattern of physical violence in the household were produced with 95% confidence intervals (CI). Women’s characteristics were described with percent distributions of women with each characteristic among each full study subsample and each of the four, mutually exclusive cohorts, defined by pattern of household violence. Differences across cohorts were tested for significance using Pearson’s chi squared test, corrected for survey design effects and converted into an F-statistic.

Multinomial logistic regressions examined associations between potential correlates and each pattern of violence in the household: 1 = physical child punishment (only); 2 = co-occurrence; and 3 = physical IPV ever (only), each compared with no violence (0 = reference category), in other words, three pairwise comparisons (0 vs. 1; 0 vs. 2; and 0 vs. 3). Associations were examined with adjusted odds ratios (aORs) and 95% CIs, with *p* < 0.05 considered statistically significant. Because surveys measured different variables, multinomial logistic regressions were carried out for a more comparable, ‘reduced’ model, adjusting for variables measured by all three surveys, with results presented in figures charted with baseline value of 1 on a logarithmic scale of base 10. Results of full models, adjusted for all variables available from each country are presented in tabular form.

Finally, to explore whether associations between physical child punishment and physical IPV remained significant after controlling for other factors, logistic regression was used to produce ORs of physical child punishment (as a dependent variable) in households affected by physical IPV (past year and before past year) compared with never using four models: 1) unadjusted; 2) adjusted for age, education, wealth and residence; 3) reduced models adjusted for factors measured by all surveys; and 4) full models, adjusted for all factors.

## Results

### Prevalence of physical violence in the household

In all countries, women reported higher levels of physical punishment of children than physical IPV ever (Table 5). Prevalence of physical child punishment only (no physical IPV ever) ranged between one fourth and one third, including 26.1% in Peru, 28.9% in Mexico and 31.9% in Colombia. Physical IPV ever only (no physical child punishment) was reported by 6.4% of women in Mexico, 11.4% in Colombia and 12.9% in Peru. Co-occurrence (both physical child punishment and physical IPV ever) was reported by 10.7% of women in Mexico, 14.4% in Peru and 15.8% in Colombia. Just over 2 in 5 women reported neither form of violence in Colombia and Peru, as did just over half of women in Mexico.

**Table 5:** Percent distribution of women by pattern of physical violence in the household

**Country**	**No violence**		**Physical child punishment (only)**		**Co-occurrence**		**Physical IPV ever (only)**	
	%	(95% CI)	%	(95% CI)	%	(95% CI)	%	(95% CI)
Colombia	40.9	(39.5–42.4)	31.9	(30.5–33.4)	15.8	(14.6–16.9)	11.4	(10.5–12.2)
Mexico	53.9	(53.2–54.7)	28.9	(28.2–29.6)	10.7	(10.3–11.2)	6.4	(6.1–6.8)
Peru	46.7	(45.2–48.2)	26.1	(24.8–27.3)	14.4	(13.3–15.4)	12.9	(11.9–13.9)

*CI* confidence interval, *IPV* intimate partner violence

### Women’s characteristics and bivariate analyses

Tables 6, 7 and 8 present percent distributions of women with each characteristic among the full study subsample and among each of four cohorts defined by pattern of violence. Bivariate analyses found significant differences across the four groups for almost all characteristics and countries, except age and residence in Colombia. Only 1.4% of women in Peru agreed with at least one reason for wife-beating, as did 3.4% of women in Colombia (not measured in Mexico). In contrast, almost one fourth (23.4%) of women in Peru (the only survey with data) agreed that physical child punishment was necessary for childrearing.

**Table 6:** Percent distribution of women with key characteristics, by pattern of violence, Colombia

**Women’s characteristics**	**Among women who reported:**				***p-*value**	**All women in study subsample**
	No violence	Physical child punishment only	Co-occurrence (both)	IPV only		
	*n* = 5,535	*n* = 4,316	*n* = 2,130	*n* = 1,537		*N* = 13,518
**Women’s age**	%	%	%	%		%
15–29 years	35.7	36.1	35.9	36.7	0.258	36.0
30–39 years	40.9	43.3	40.4	43.8		41.9
40–49 years	23.4	20.6	23.8	19.5		22.1
**Women’s education**						
Primary or none	22.3	22.6	27.4	21.8	** < 0.001**	23.1
Lower secondary	10.3	10.3	15.1	15.0		11.6
Upper secondary	33.8	34.4	36.7	37.3		34.8
Post-secondary	33.7	32.7	20.8	25.9		30.5
**Household wealth**						
Poorest	35.0	36.4	41.4	39.3	**0.005**	36.9
Middle	32.6	32.6	35.0	34.0		33.1
Richest	32.5	30.9	23.6	26.6		29.9
**Residence**						
Urban	74.1	72.2	72.7	73.6	0.522	73.2
Rural	25.9	27.8	27.3	26.4		26.8
**Indigenous ethnicity**						
No	92.4	94.6	93.7	92.2	**0.002**	93.3
Yes	7.6	5.4	6.3	7.8		6.7
**Married/mother < age 18**						
No	65.5	64.7	54.3	52.4	**< 0.001**	62.0
Yes	34.5	35.3	45.7	47.6		38.0
**Number of children aged 1–14**						
1	58.3	50.4	45.7	58.0	** < 0.001**	53.8
2+	41.7	49.6	54.3	42.0		46.2
**Age of youngest child**						
1 year	13.4	10.6	11.0	9.8	** < 0.001**	11.8
2–5 years	36.2	43.5	42.1	34.9		39.3
6–9 years	24.6	26.9	27.2	28.3		26.2
10–14 years	25.7	19.0	19.6	27.0		22.8
**Partner tries to socially isolate her**						
No	90.8	90.5	56.7	60.7	** < 0.001**	81.9
Yes	9.2	9.5	43.3	39.3		18.1
**Partner drinks to excess**	*Not measured*					
**Joint money decisions**						
No	54.2	58.0	63.0	68.2	**< 0.001**	58.4
Yes	45.8	42.0	37.0	31.8		41.6
**Shared household chores**						
No	61.5	67.2	74.5	74.3	**< 0.001**	66.8
Yes	38.5	32.8	25.5	25.7		33.2
**Wife beating justified**						
No/DK 5 reasons	96.8	96.9	95.3	96.2	**0.031**	96.6
Yes (1+ of 5 reason)	3.2	3.1	4.7	3.8		3.4
**Physical punishment necessary**	*Not measured*					
**Violence in her childhood**						
None	37.2	18.1	13.7	28.8	** < 0.001**	26.4
By caregiver only	34.6	47.9	35.2	32.5		38.7
Co-occurrence	19.5	28.8	44.0	26.9		27.2
Exposure to IPV	8.7	5.1	7.2	11.9		7.7
**Violence in partner’s childhood**						
None/DK	69.9	60.0	39.9	48.5	** < 0.001**	59.6
By caregiver only	13.7	17.5	20.1	15.8		16.1
Co-occurrence	8.8	13.7	26.9	21.5		14.7
Exposure to IPV	7.7	8.7	13.1	14.2		9.6

*DK* Don’t know, *IPV* intimate partner violence, significant *p-*values are bolded. All figures are weighted

**Table 7:** Percent distribution of women with key characteristics, by pattern of violence, Mexico

**Women’s characteristics**	**Among women who reported:**				***p-*value**	**All women in study subsample**
	**No violence**	**Physical child punishment only**	**Co-occurrence (both)**	**IPV only**		
	*n* = 20,551	*n* = 11,013	*n* = 4,085	*n* = 2,448		*N* = 38,097
**Women’s age**	%	%	%	%		%
15–29 years	34.4	29.1	30.1	32.3	** < 0.001**	32.3
30–39 years	41.5	45.7	46.3	42.6		43.3
40–49 years	24.1	25.2	23.6	25.1		24.4
**Women’s education**						
Primary or none	22.2	22.3	28.2	26.7	** < 0.001**	23.2
Lower secondary	36.7	39.7	42.3	44.3		38.7
Upper secondary	22.9	22.4	20.4	18.6		22.2
Post-secondary	18.2	15.5	9.1	10.4		16.0
**Household wealth**						
Poorest	36.8	38.9	45.6	44.1	** < 0.001**	38.8
Middle	32.2	33.4	33.6	33.0		32.8
Richest	31.0	27.6	20.8	23.0		28.4
**Residence**						
Urban	50.0	46.2	48.3	51.3	** < 0.001**	48.8
Semi-urban	23.7	26.7	25.3	22.3		24.6
Rural	26.3	27.1	26.4	26.5		26.6
**Indigenous ethnicity**						
No	92.1	93.9	91.0	90.5	** < 0.001**	92.4
Yes	7.9	6.1	9.0	9.5		7.6
**Married/mother < age 18**						
No	71.5	71.3	58.5	61.1	** < 0.001**	69.4
Yes	28.5	28.7	41.5	38.9		30.6
**Number of children aged 1–14**						
1	44.9	32.5	31.9	40.5	** < 0.001**	39.6
2+	55.1	67.5	68.1	59.5		60.4
**Age of youngest child**						
1 year	13.6	9.7	8.7	11.5	** < 0.001**	11.8
2–5 years	38.2	41.8	39.2	36.5		39.3
6–9 years	24.7	28.1	28.5	24.9		26.1
10–14 years	23.5	20.4	23.7	27.0		22.9
**Partner tries to socially isolate her**						
No	95.9	91.3	66.0	72.9	** < 0.001**	89.9
Yes	4.1	8.7	34.0	27.1		10.1
**Partner drinks to excess**						
No	83.3	74.3	47.4	53.8	** < 0.001**	74.9
Yes	16.7	25.7	52.6	46.2		25.1
**Joint money decisions**						
No	44.2	47.6	60.7	57.4	** < 0.001**	47.8
Yes	55.8	52.4	39.3	42.6		52.2
**Shared household chores**						
No	80.1	80.8	87.3	86.4	** < 0.001**	81.5
Yes	19.9	19.2	12.7	13.6		18.5
**Wife beating justified**	*Not measured*					
**Physical punishment necessary**	*Not measured*					
**Violence in her childhood**						
None	69.5	41.0	24.6	41.1	** < 0.001**	54.6
By caregiver only	11.1	27.5	22.7	16.0		17.4
Co-occurrence	9.9	20.3	39.6	26.1		17.2
Exposure to IPV	9.5	11.2	13.1	16.9		10.8
**Violence in partner’s childhood**						
None/DK	73.4	53.4	33.1	45.4	** < 0.001**	61.5
By caregiver only	11.5	22.4	22.2	17.5		16.2
Co-occurrence	8.9	14.8	31.6	25.5		14.1
Exposure to IPV	6.2	9.4	13.1	11.6		8.2

*DK* Don’t know, *IPV* intimate partner violence; significant *p-*values are bolded. All figures are weighted

**Table 8:** Percent distribution of women with key characteristics, by pattern of violence, Peru

**Women’s characteristics**	**Among women who reported:**				***p*-value**	**All women in study subsample**
	**No violence**	**Physical child punishment only**	**Co-occurrence (both)**	**IPV only**		
	*n* = 8,009	*n* = 4,472	*n* = 2,462	*n* = 2,213		*N* = 17,156
**Women’s age**	%	%	%	%		%
15–29 years	28.6	33.6	29.1	22.7	** < 0.001**	29.2
30–39 years	42.9	43.2	45.3	44.4		43.5
40–49 years	28.6	23.2	25.6	32.8		27.3
**Women's education**						
Primary or none	21.6	28.5	28.3	25.7	**0.001**	24.9
Lower secondary	10.7	12.3	14.2	14.9		12.2
Upper secondary	29.6	29.3	30.6	31.8		30.0
Post-secondary	38.1	30.0	26.9	27.6		33.0
**Household wealth**						
Poorest	35.9	42.1	44.6	40.8	** < 0.001**	39.4
Middle	32.7	32.0	32.8	37.3		33.2
Richest	31.4	25.8	22.6	21.9		27.5
**Residence**						
Urban	76.8	72.8	74.0	78.8	** < 0.001**	75.6
Rural	23.2	27.2	26.0	21.2		24.4
**Indigenous ethnicity**						
No	82.0	85.2	77.7	73.8	** < 0.001**	81.2
Yes	18.0	14.8	22.3	26.2		18.9
**Married/mother < age 18**						
No	73.5	68.4	64.4	64.8	** < 0.001**	69.8
Yes	26.5	31.6	35.6	35.2		30.2
**Number of children aged 1–14**						
1	52.5	37.3	35.0	46.3	** < 0.001**	45.2
2+	47.5	62.7	65.0	53.7		54.8
**Age of youngest child**						
1 year	13.6	10.7	9.7	9.3	** < 0.001**	11.8
2–5 years	42.4	56.4	51.6	41.5		47.3
6–9 years	26.5	24.4	26.3	29.5		26.3
10–14 years	17.5	8.4	12.5	19.6		14.7
**Partner tries to socially isolate her**						
No	95.4	92.9	74.2	74.5	** < 0.001**	89.0
Yes	4.6	7.1	25.8	25.5		11.0
**Partner drinks to excess**						
No	47.5	42.3	23.2	30.1	** < 0.001**	40.4
Yes	52.5	57.7	76.8	69.9		59.6
**Joint money decisions**						
No	69.5	72.5	78.1	77.0	** < 0.001**	72.5
Yes	30.5	27.5	21.9	23.0		27.5
**Shared household chores**	*Not measured*					
**Wife beating justified**						
No/DK all reasons	99.0	98.0	98.0	98.7	**0.003**	98.6
Yes (1+ of 5 reasons)	1.0	2.0	2.0	1.3		1.4
**Physical punishment necessary**						
No/DK	88.3	61.1	59.6	85.0	** < 0.001**	76.6
Yes	11.7	38.9	40.4	15.0		23.4
**Violence in her childhood**						
None	33.6	15.5	11.1	18.5	** < 0.001**	23.7
By caregiver only	32.2	43.6	33.7	27.0		34.7
Co-occurrence	24.1	34.0	46.7	44.6		32.6
Exposure to IPV	10.1	6.9	8.4	9.9		9.0
**Violence in partner's childhood**	*Not measured*					

*DK* Don’t know, *IPV* intimate partner violence; significant *p-*values are bolded. All figures are weighted

### Correlates of physical violence in the household

**Fig. 1:**
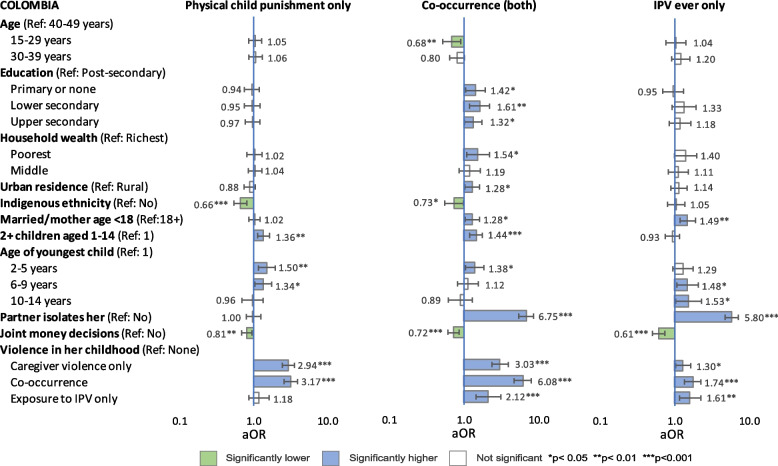
Reduced model: Adjusted odds ratios of patterns of physical violence, Colombia

**Fig. 2:**
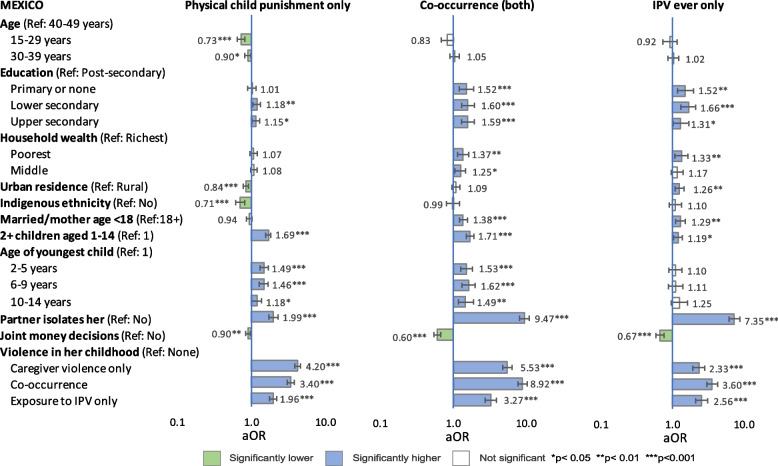
Reduced model: Adjusted odds ratios of patterns of physical violence, Mexico

**Fig. 3:**
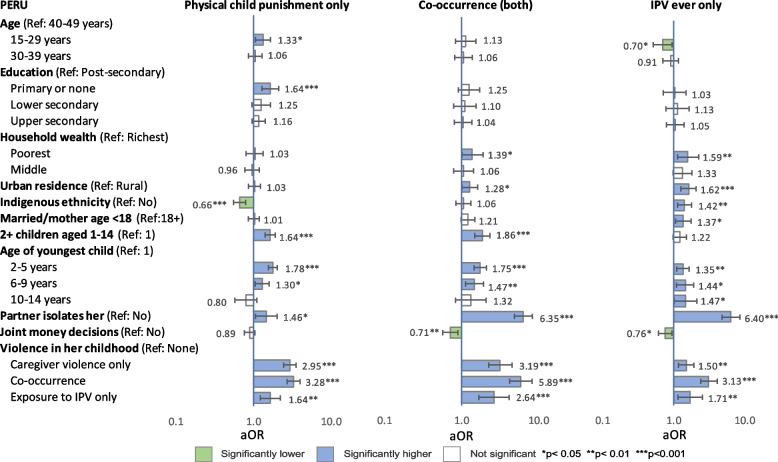
Reduced model: Adjusted odds ratios of patterns of physical violence, Peru

Multinomial regression analyses examined associations between possible correlates and three mutually exclusive patterns of household violence: 1) physical child punishment (only), 2) co-occurring physical child punishment and physical IPV, and 3) physical IPV (only) –each compared with ‘no violence’, after adjusting for other variables. Results of reduced models (limited to variables measured by all three countries) are presented in Figs. 1, 2 and 3 and Supplemental Table B. Results of full models are presented in Tables 9, 10 and 11. Supplemental Table C presents unadjusted and adjusted odds ratios (from both models) of co-occurrence side by side.

**Table 9:** Full Model: Odds ratios of each pattern of violence, adjusted for all variables, Colombia

**Women’s characteristics**	**Physical punishment (only)**			**Co-occurrence (both)**			**Physical IPV ever (only)**		
	**aOR**	**CI (95%)**	***p*-value**	**aOR**	**CI (95%)**	***p*-value**	**aOR**	**CI (95%)**	***p*-value**
**Age** (Ref: 40–49 years)									
15–29 years	1.06	(0.87–1.31)	0.555	**0.69**	**(0.51–0.94)**	**0.018**	1.05	(0.76–1.44)	0.781
30–39 years	1.07	(0.87–1.31)	0.536	0.79	(0.61–1.02)	0.065	1.18	(0.90–1.56)	0.236
**Education** (Ref: Post-secondary)									
Primary or none	0.92	(0.73–1.16)	0.480	1.35	(0.98–1.85)	0.063	0.89	(0.64–1.23)	0.482
Lower secondary	0.94	(0.73–1.20)	0.598	**1.52**	**(1.11–2.10)**	**0.010**	1.26	(0.87–1.83)	0.219
Upper secondary	0.96	(0.76–1.20)	0.693	1.29	(0.98–1.69)	0.071	1.13	(0.82–1.56)	0.446
**Household wealth** (Ref: Richest)									
Poorest	1.00	(0.79–1.26)	0.999	**1.54**	**(1.09–2.18)**	**0.013**	**1.40**	**(1.00–1.95)**	**0.048**
Middle	1.03	(0.84–1.27)	0.750	1.20	(0.86–1.67)	0.280	1.13	(0.84–1.53)	0.426
**Urban residence** (Ref: Rural)	0.89	(0.74–1.06)	0.186	**1.31**	**(1.04–1.66)**	**0.021**	1.16	(0.90–1.49)	0.261
**Indigenous ethnicity** (Ref: No)	**0.68**	**(0.55–0.83)**	** < 0.001**	**0.74**	**(0.56–0.99)**	**0.044**	1.08	(0.82–1.42)	0.570
**Married/mother < 18** (Ref:18+)	1.02	(0.86–1.21)	0.800	**1.28**	**(1.04–1.58)**	**0.022**	**1.49**	**(1.18–1.88)**	**0.001**
**2+ children aged 1–14** (Ref: 1)	**1.39**	**(1.15–1.67)**	**0.001**	**1.51**	**(1.23–1.86)**	** < 0.001**	0.96	(0.78–1.20)	0.744
**Age youngest eligible child** (Ref: 1)									
2–5 years	**1.51**	**(1.16–1.96)**	**0.002**	**1.39**	**(1.04–1.87)**	**0.027**	1.29	(0.95–1.77)	0.107
6–9 years	**1.35**	**(1.04–1.74)**	**0.023**	1.14	(0.82–1.60)	0.429	**1.49**	**(1.07–2.09)**	**0.019**
10–14 years	0.98	(0.71–1.34)	0.885	0.92	(0.63–1.34)	0.656	**1.58**	**(1.06–2.36)**	**0.024**
**Partner isolates her** (Ref: No)	0.92	(0.72–1.16)	0.462	**5.72**	**(4.48–7.31)**	** < 0.001**	**5.03**	**(4.09–6.20)**	** < 0.001**
**Partner drinks to excess**	*Not measured*								
**Joint money decisions** (Ref: No)	**0.83**	**(0.71–0.97)**	**0.021**	**0.77**	**(0.64–0.93)**	**0.006**	**0.65**	**(0.54–0.78)**	** < 0.001**
**Shared chores** (Ref: No)	**0.81**	**(0.67–0.97)**	**0.022**	**0.70**	**(0.56–0.88)**	**0.002**	**0.68**	**(0.55–0.85)**	**0.001**
**Wife beating justified** (Ref: No/DK)	1.01	(0.75–1.35)	0.953	1.40	(0.97–2.02)	0.072	1.04	(0.68–1.59)	0.845
**Physical punishment needed**	*Not measured*								
**Violence in her childhood** (Ref: No)									
Caregiver violence only	**2.86**	**(2.37–3.46)**	** < 0.001**	**2.91**	**(2.24–3.78)**	** < 0.001**	**1.26**	**(1.00–1.59)**	**0.048**
Co-occurrence	**2.99**	**(2.42–3.70)**	** < 0.001**	**5.20**	**(3.93–6.88)**	** < 0.001**	**1.55**	**(1.21–1.99)**	**0.001**
Exposure to IPV only	1.12	(0.82–1.53)	0.474	**1.83**	**(1.22–2.74)**	**0.003**	**1.44**	**(1.04–1.99)**	**0.030**
**Violence in partner's childhood** (Ref: No/DK)									
Caregiver violence only	**1.41**	**(1.08–1.84)**	**0.012**	**2.14**	**(1.63–2.81)**	** < 0.001**	**1.41**	**(1.01–1.96)**	**0.045**
Co-occurrence	**1.71**	**(1.33–2.21)**	** < 0.001**	**3.98**	**(3.14–5.04)**	** < 0.001**	**2.78**	**(2.11–3.66)**	** < 0.001**
Exposure to IPV only	1.23	(0.90–1.69)	0.195	**2.49**	**(1.82–3.39)**	** < 0.001**	**2.24**	**(1.55–3.25)**	** < 0.001**

*aOR* adjusted odds ratios, *CI* Confidence interval, *DK* doesn't know, *IPV* intimate partner violence, *Ref* reference category; significant *p*-values are bolded

**Table 10:** Full Model: Odds ratios of each pattern of violence, adjusted for all variables, Mexico

**Women’s characteristics**	**Physical punishment (only)**			**Co-occurrence (both)**			**Physical IPV ever (only)**		
	**aOR**	**CI (95%)**	***p*-value**	**aOR**	**CI (95%)**	***p*-value**	**aOR**	**CI (95%)**	***p*-value**
**Age** (Ref: 40–49 years)									
15–29 years	**0.73**	**(0.65–0.82)**	** < 0.001**	0.84	(0.69–1.02)	0.078	0.92	(0.73–1.16)	0.488
30–39 years	0.91	(0.82–1.00)	0.051	1.08	(0.93–1.26)	0.309	1.05	(0.88–1.25)	0.591
**Education** (Ref: Post-secondary)									
Primary or none	1.01	(0.89–1.15)	0.879	**1.35**	**(1.07–1.72)**	**0.012**	**1.36**	**(1.04–1.76)**	**0.022**
Lower secondary	**1.15**	**(1.03–1.29)**	**0.017**	**1.39**	**(1.13–1.71)**	**0.002**	**1.47**	**(1.15–1.87)**	**0.002**
Upper secondary	1.12	(0.99–1.26)	0.065	**1.38**	**(1.12–1.70)**	**0.002**	1.18	(0.91–1.52)	0.218
**Household wealth** (Ref: Richest)									
Poorest	1.03	(0.92–1.16)	0.610	1.14	(0.95–1.38)	0.162	1.15	(0.93–1.42)	0.209
Middle	1.05	(0.95–1.16)	0.335	1.12	(0.94–1.33)	0.201	1.07	(0.89–1.30)	0.465
**Residence** (Ref: Rural)									
Urban	**0.87**	**(0.79–0.97)**	**0.011**	**1.22**	**(1.04–1.43)**	**0.015**	**1.33**	**(1.11–1.60)**	**0.002**
Semi-urban	1.05	(0.94–1.17)	0.377	1.15	(0.98–1.34)	0.089	1.04	(0.87–1.24)	0.647
**Indigenous ethnicity** (Ref: No)	**0.73**	**(0.63–0.84)**	** < 0.001**	1.03	(0.83–1.27)	0.803	1.12	(0.91–1.38)	0.285
**Married/mother < 18** (Ref:18+)	0.93	(0.85–1.01)	0.089	**1.34**	**(1.17–1.53)**	** < 0.001**	**1.26**	**(1.08–1.48)**	**0.004**
**2+ children aged 1–14** (Ref: 1)	**1.68**	**(1.55–1.81)**	** < 0.001**	**1.70**	**(1.50–1.92)**	** < 0.001**	**1.18**	**(1.02–1.36)**	**0.029**
**Age of youngest eligible child** (Ref: 1)									
2–5 years	**1.52**	**(1.34–1.72)**	** < 0.001**	**1.62**	**(1.33–1.98)**	** < 0.001**	1.16	(0.94–1.44)	0.172
6–9 years	**1.48**	**(1.29–1.70)**	** < 0.001**	**1.68**	**(1.35–2.10)**	** < 0.001**	1.15	(0.91–1.45)	0.236
10–14 years	**1.19**	**(1.02–1.38)**	**0.026**	**1.56**	**(1.22–2.01)**	** < 0.001**	1.30	(1.00–1.70)	0.052
**Partner isolates her** (Ref: No)	**1.74**	**(1.51–2.00)**	** < 0.001**	**6.76**	**(5.74–7.94)**	** < 0.001**	**5.44**	**(4.55–6.51)**	** < 0.001**
**Partner drinks to excess** (Ref: No)	**1.45**	**(1.33–1.59)**	** < 0.001**	**3.35**	**(2.97–3.78)**	** < 0.001**	**2.84**	**(2.47–3.26)**	** < 0.001**
**Joint money decisions** (Ref: No)	**0.93**	**(0.86–1.00)**	**0.049**	**0.67**	**(0.60–0.76)**	** < 0.001**	**0.73**	**(0.64–0.84)**	** < 0.001**
**Shared chores** (Ref: No)	0.98	(0.89–1.07)	0.662	**0.74**	**(0.64–0.87)**	** < 0.001**	**0.77**	**(0.63–0.94)**	**0.010**
**Wife beating justified**	*Not measured*								
**Physical punishment needed**	*Not measured*								
**Violence in her childhood** (Ref: No)									
Caregiver violence only	**3.63**	**(3.30–3.99)**	** < 0.001**	**4.33**	**(3.70–5.06)**	** < 0.001**	**1.95**	**(1.61–2.36)**	** < 0.001**
Co-occurrence	**2.85**	**(2.55–3.18)**	** < 0.001**	**6.28**	**(5.37–7.35)**	** < 0.001**	**2.74**	**(2.29–3.28)**	** < 0.001**
Exposure to IPV only	**1.72**	**(1.53–1.94)**	** < 0.001**	**2.46**	**(2.04–2.97)**	** < 0.001**	**2.03**	**(1.68–2.47)**	** < 0.001**
**Violence in partner’s childhood** (Ref: No/DK)									
Caregiver violence only	**1.88**	**(1.70–2.07)**	** < 0.001**	**2.45**	**(2.09–2.86)**	** < 0.001**	**1.70**	**(1.40–2.07)**	** < 0.001**
Co-occurrence	**1.58**	**(1.40–1.79)**	** < 0.001**	**3.62**	**(3.09–4.24)**	** < 0.001**	**2.65**	**(2.21–3.18)**	** < 0.001**
Exposure to IPV only	**1.64**	**(1.42–1.89)**	** < 0.001**	**2.67**	**(2.22–3.21)**	** < 0.001**	**1.95**	**(1.57–2.42)**	** < 0.001**

aOR: adjusted odds ratios; CI: Confidence interval; DK: doesn't know; IPV: intimate partner violence; Ref: reference category; significant *p*-values are bolded

**Table 11:** Full Model: Odds ratios of each pattern of violence. adjusted for all variables, Peru

**Women’s characteristics**	**Physical punishment (only)**			**Co-occurrence (both)**			**Physical IPV ever (only)**		
	**aOR**	**CI (95%)**	***p*-value**	**aOR**	**CI (95%)**	***p*-value**	**aOR**	**CI (95%)**	***p*-value**
**Age** (Ref: 40–49 years)									
15–29 years	**1.37**	**(1.09–1.74)**	**0.008**	1.11	(0.81–1.51)	0.527	**0.67**	**(0.49–0.91)**	**0.011**
30–39 years	1.08	(0.87–1.34)	0.472	1.06	(0.80–1.40)	0.693	0.88	(0.68–1.14)	0.332
**Education** (Ref: Post-secondary)									
Primary or none	**1.87**	**(1.44–2.42)**	** < 0.001**	**1.44**	**(1.03–2.01)**	**0.034**	1.06	(0.73–1.53)	0.774
Lower secondary	**1.43**	**(1.07–1.92)**	**0.016**	1.27	(0.90–1.81)	0.179	1.12	(0.77–1.64)	0.553
Upper secondary	**1.28**	**(1.04–1.58)**	**0.018**	1.14	(0.87–1.50)	0.339	1.03	(0.78–1.37)	0.818
**Household wealth** (Ref: Richest)									
Poorest	0.99	(0.76–1.29)	0.944	1.30	(0.94–1.81)	0.111	**1.57**	**(1.11–2.22)**	**0.011**
Middle	0.94	(0.75–1.17)	0.566	1.01	(0.74–1.37)	0.950	1.30	(0.96–1.77)	0.092
**Urban residence** (Ref: Rural)	1.08	(0.90–1.30)	0.389	**1.41**	**(1.11–1.79)**	**0.005**	**1.70**	**(1.33–2.18)**	** < 0.001**
**Indigenous ethnicity** (Ref: No)	**0.63**	**(0.52–0.75)**	** < 0.001**	0.96	(0.77–1.21)	0.753	**1.36**	**(1.09–1.70)**	**0.007**
**Married/mother < 18** (Ref:18+)	1.00	(0.85–1.18)	0.974	1.18	(0.95–1.46)	0.133	**1.38**	**(1.07–1.77)**	**0.013**
**2+ children aged 1–14** (Ref: 1)	**1.54**	**(1.31–1.81)**	** < 0.001**	**1.73**	**(1.36–2.21)**	** < 0.001**	1.22	(0.98–1.51)	0.072
**Age of youngest eligible child** (Ref: 1)									
2–5 years	**1.76**	**(1.53–2.02)**	** < 0.001**	**1.74**	**(1.43–2.10)**	** < 0.001**	**1.33**	**(1.08–1.63)**	**0.006**
6–9 years	**1.27**	**(1.03–1.57)**	**0.027**	**1.42**	**(1.07–1.88)**	**0.014**	**1.45**	**(1.09–1.92)**	**0.011**
10–14 years	0.75	(0.53–1.05)	0.093	1.20	(0.75–1.93)	0.438	**1.45**	**(1.01–2.08)**	**0.045**
**Partner isolates her** (Ref: No)	**1.46**	**(1.04–2.04)**	**0.028**	**6.00**	**(4.45–8.08)**	** < 0.001**	**6.09**	**(4.53–8.18)**	** < 0.001**
**Partner drinks to excess** (Ref: No)	1.10	(0.95–1.28)	0.201	**2.47**	**(1.96–3.12)**	** < 0.001**	**1.88**	**(1.50–2.36)**	** < 0.001**
**Joint money decisions** (Ref: No)	0.92	(0.78–1.08)	0.303	**0.74**	**(0.59–0.95)**	**0.016**	**0.77**	**(0.62-0.96)**	**0.019**
**Shared chores**	*Not measured*								
**Wife beating justified** (Ref: No/DK)	1.49	(0.88–2.52)	0.138	**1.75**	**(1.04–2.94)**	**0.034**	1.46	(0.80–2.65)	0.219
**Phys. punish. needed** (Ref: No/DK)	**4.53**	**(3.77–5.45)**	** < 0.001**	**4.50**	**(3.55–5.70)**	** < 0.001**	1.21	(0.92–1.59)	0.176
**Violence in her childhood** (Ref: No)									
Caregiver violence only	**2.57**	**(2.11–3.12)**	** < 0.001**	**2.67**	**(1.87–3.83)**	** < 0.001**	**1.46**	**(1.13–1.87)**	**0.003**
Co-occurrence	**2.93**	**(2.39–3.60)**	** < 0.001**	**5.12**	**(3.64–7.20)**	** < 0.001**	**3.02**	**(2.31–3.96)**	** < 0.001**
Exposure to IPV only	**1.68**	**(1.26–2.24)**	** < 0.001**	**2.62**	**(1.67–4.09)**	** < 0.001**	**1.69**	**(1.15–2.50)**	**0.008**
**Violence in partner’s childhood** (Ref: No/DK)									
Caregiver violence only	*Not measured*								
Co-occurrence	*Not measured*								
Exposure to IPV only	*Not measured*								

*aOR* adjusted odds ratios, *CI* Confidence interval, *DK* doesn’t know, *IPV* intimate partner violence, *Phys. punish.* Physical punishment, *Ref* reference category; significant *p*-values are bolded

#### Sociodemographic factors

Associations between sociodemographic factors and patterns of violence were inconsistent across countries. In both full and reduced models, younger age was significantly associated with lower odds of co-occurrence in Colombia, physical child punishment (only) in Mexico, and IPV (only) in Peru, and with higher odds of physical child punishment (only) in Peru. Age was not significantly correlated with other patterns of violence.

In full models from all countries, lower education was significantly associated with higher odds of co-occurrence compared with post-secondary, but specific levels that were significant varied by country, and the relationship between rising education level and declining odds of co-occurrence was not always linear. Associations between education and other patterns of violence were inconsistent across countries and models.

In reduced models, the poorest wealth tercile was significantly correlated with co-occurrence in all countries, but in full models, wealth was not associated with any pattern of violence except co-occurrence among the poorest women in Colombia and IPV (only) among the poorest women in Colombia and Peru.

After adjusting for all factors in full models, urban (compared with rural) residence was associated with significantly higher odds of co-occurrence in all countries and of IPV (only) in Mexico and Peru (but not Colombia). In contrast, urban residence was associated with significantly lower odds of physical child punishment (only) in Mexico (both models); but not significant in Colombia or Peru in any model.

#### Indigenous ethnicity

After adjusting for other factors (full and reduced models), indigenous ethnicity was protective —meaning that compared with other women, women identified as indigenous had significantly lower odds— for co-occurrence in Colombia (but not Mexico or Peru) and for physical child punishment (only) in all three countries. In contrast, odds of IPV (only) were elevated among indigenous women in all countries, but significantly so only in Peru. Sensitivity testing in Mexico and Peru using indigenous ethnicity defined by culture/ancestors did not change these findings.

#### Reproductive history

In full and reduced models, aORs of co-occurrence were significantly elevated among women who had a history of child marriage/early motherhood (compared with women who did not) in Colombia and Mexico, but not Peru. In all countries, women’s history of child marriage/early motherhood was significantly associated with IPV (only) but not physical child punishment (only). Having more than one child aged 1–14 in the household was significantly associated with higher odds of all patterns of violence in all countries and models except IPV (only) in Colombia and Peru.

In full and reduced models, having a child aged 2–5 was significantly associated with co-occurrence and physical child punishment (only) across all countries, and with IPV (only) in Peru. In almost all countries and models, aORs of co-occurrence and physical child punishment (only) declined as age of youngest child rose, while aORs of IPV (only) rose, but associations were not always significant.

#### Partnership dynamics

In all countries, aORs of both co-occurrence and IPV (only) were 5–7 times higher among women whose partner tried to socially isolate them than among other women. Social isolation was also significantly associated with higher odds physical child punishment (only) in Mexico and Peru (aOR of 1.74 and 1.46 respectively) but not Colombia.

In Mexico and Peru, partner’s excess drinking was significantly associated with all patterns of violence except physical child punishment (only) in Peru. However, the association with physical child punishment (only) did become significant (aOR 1.17; 95% CI: 1.02–1.35, *p* = 0.026) in Peru when the model was run without agreement with physical child punishment, suggesting some covariance. When the full model was run with frequency of partner’s drunkenness in Peru, aORs of co-occurrence were 2.22 aOR (95% CI: 1.76–2.82, *p* < 0.001) for ‘sometimes’ (versus never) drunk and 14.9 aOR (95% CI: 8.90–25.0, *p* < 0.001) for ‘frequently’ drunk.

In all models and countries, collaborative partnership dynamics, meaning joint financial decision-making (all countries) and shared domestic chores (Colombia and Mexico), were significantly protective for most patterns of violence, except shared chores and physical child punishment (only) in Mexico, and joint decision-making and physical child punishment (only) in Peru. Shared chores and joint decision-making covaried to some degree however, and their protective effect was muted when both were included in models, as seen by comparing full and reduced models in Colombia and Mexico.

#### Social norms

After controlling for other factors, agreement with wife-beating was not significantly associated with any pattern of violence in Colombia and Peru except co-occurrence in Peru (not measured in Mexico). However, in Colombia, when the ‘pattern of household violence’ variable was constructed with severe physical punishment of children (beating with objects) instead of any physical punishment, support for wife-beating was significantly associated with severe child punishment (only), 1.45 aOR (95% CI: 1.07-1.97, *p* = 0.017) and with co-occurring severe child punishment and IPV (aOR 1.53, 95% CI: 1.04–2.26, *p* = 0.031). The association with IPV only (no severe child punishment) remained insignificant.

In Peru, agreement with the necessity of physical child punishment was significantly associated with both co-occurrence and physical child punishment (only). The aORs of both these patterns of violence were more than four times higher among women who agreed with physical punishment than among those who did not. In contrast, the slightly elevated aOR of IPV (only) among women who agreed (vs. disagreed) was not significant.

#### Histories of childhood violence

In all countries and models, aORs of co-occurrence in the current household were more than five times higher among women who reported co-occurrence in childhood compared with those who did not. In fact, in all models and countries, almost all patterns of violence (caregiver violence only, co-occurrence and exposure to IPV only) in either respondents’ or partners’ childhoods were significantly correlated with elevated odds of almost all patterns of violence in the current household. Exceptions were in Peru, which did not measure violence in the partner’s childhood, and in Colombia, where histories of childhood exposure to IPV (only) among respondents or partners were not significantly associated with physical child punishment (only) in the current household.

Despite efforts to limit covariance among variables, some interactions remained, particularly between respondents’ and partners’ histories of violence in childhood. When these two variables were replaced with a consolidated variable (available only from Colombia and Mexico), aORs of co-occurrence in the current household were around 20 times higher among women who reported co-occurrence in both their own and their partner’s childhoods compared with women who reported no violence in either their own or their partner’s childhoods, with 21.60 aOR (95% CI: 14.32–32.56, *p* < 0.001) in Colombia and 20.71 aOR (95% CI: 16.06–26.70, *p* < 0.001) in Mexico.

### Correlations between physical child punishment and IPV

When associations between physical child punishment (a dependent variable) and physical IPV (an independent variable) were examined with logistic regression, odds of physical child punishment remained significantly elevated (*p* < 0.001) in households affected by IPV (past year and before past year compared with never) across all countries and models (Table 12). Odds ratios of physical child punishment in households affected by IPV declined slightly as more variables were added but remained about 1.5 to 2 (*p* < 0.001) in all countries even after adjusting for all factors in the full model.

**Table 12:** Odds ratios of physical child punishment in households affected by intimate partner violence

**IPV Timeframe**	**Colombia**			**Mexico**			**Peru**		
	**OR**	**(95% CI)**	***p*-value**	**OR**	**(95% CI)**	***p*-value**	**OR**	**(95% CI)**	***p*-value**
	**Unadjusted odds ratios**								
Never	Ref			Ref			Ref		
Past year	1.83	(1.58–2.12)	< 0.001	3.67	(3.26–4.12)	< 0.001	2.35	(1.95–2.84)	< 0.001
Before past year	1.72	(1.41–2.11)	< 0.001	2.62	(2.35–2.93)	< 0.001	1.79	(1.53–2.10)	< 0.001
	**Adjusted for age, education, wealth and residence only**								
Never	Ref			Ref			Ref		
Past year	1.81	(1.56–2.09)	< 0.001	3.68	(3.27–4.14)	< 0.001	2.31	(1.90–2.80)	< 0.001
Before past year	1.71	(1.40–2.09)	< 0.001	2.54	(2.27–2.83)	< 0.001	1.82	(1.55–2.14)	< 0.001
	**Reduced model adjusted for factors available from all three countries**								
Never	Ref			Ref			Ref		
Past year	1.66	(1.40–1.97)	< 0.001	2.38	(2.08–2.72)	< 0.001	2.08	(1.63–2.65)	< 0.001
Before past year	1.56	(1.30–1.86)	< 0.001	1.94	(1.72–2.19)	< 0.001	1.65	(1.39–1.96)	< 0.001
	**Full model adjusted for all factors**								
Never	Ref			Ref			Ref		
Past year	1.56	(1.31–1.85)	< 0.001	2.03	(1.77–2.32)	< 0.001	1.94	(1.50–2.52)	< 0.001
Before past year	1.46	(1.22–1.74)	< 0.001	1.72	(1.52–1.94)	< 0.001	1.58	(1.32–1.90)	< 0.001

*CI* confidence interval, *IPV* intimate partner violence, *OR* odds ratio, *Ref* reference category

## Discussion

This is the first multi-country study from LAC to investigate correlates of co-occurring physical child punishment and physical IPV in the same household using national, population-based datasets. In all three countries, co-occurrence was significantly associated with women’s lower education, urban residence, child marriage/early motherhood, 2+ children in the household, a child aged 2–5, a partner who tried to isolate her from family or friends, and a history of violence (caregiver violence, exposure to IPV or both) in her own childhood. Significant associations were also found with partner’s excess drinking (Mexico and Peru), women’s agreement with physical child punishment (Peru) and partner’s history of childhood violence (Colombia and Mexico). These findings generally harmonize with previous research on violent discipline, IPV and their intersection [21, 23, 25, 58, 64, 65, 69–71, 74, 79]. Correlations with lower education, partner’s drinking, social isolation and support for violence against children are consistent with some, though not all previous studies of co-occurrence in LMICs [25] and high-income settings [21].

Conversely, collaborative partnership dynamics, including shared household decision-making (all countries) and shared household chores (Colombia and Mexico) were significantly protective. There is a dearth of quantitative studies from LMICs examining co-occurrence and partnership dynamics; however, these findings echo qualitative research from Uganda linking co-occurrence with patriarchal family structures that normalize women’s subordinate position and encourage violence to enforce power structures within families [46].

The study did not find consistent evidence that socio-demographic characteristics (age, education, wealth, residence or ethnicity) were shared risk factors for physical violence in the household across the three countries. In fact, indigenous ethnicity and urban residence tended to be protective for physical child punishment but a risk factor for IPV, though associations were not always significant. This finding echoes the separate literatures on IPV [70] and violent discipline, including evidence suggesting that poverty may not be a universal correlate of household violence [29, 40, 41, 100, 101]. Similarly, child marriage/early motherhood was significantly associated with IPV (only) in all countries and with co-occurrence in Mexico and Peru (consistent with the IPV literature [80]), but not with physical child punishment (only) in any country.

The study found evidence that partner’s excess drinking was a significant shared risk factor for all three patterns of violence in Mexico. In Peru it was a shared risk factor after excluding attitudes towards physical child punishment, with which it covaried. These findings support a large literature theorizing that harmful use of alcohol may be a policy-relevant risk factor for both IPV and violence against children [23, 64–66].

A partner who tried to isolate her from family or friends was a shared risk factor for all patterns of violence except physical child punishment (only) in Colombia, while collaborative partnership dynamics, such as shared financial decision-making (all countries) and shared domestic chores (Colombia and Mexico) were protective. These findings are consistent with theories articulated by major international frameworks for violence prevention that greater gender equality and empowerment of women within households may protect women and children from violence [82, 83]. They also echo evidence from the IPV literature that joint decision-making may be protective for IPV against women even more than any female decision-making [63, 65, 87].

This study did not find consistent evidence that social norms justifying violence were shared risk factors, after controlling for other factors. Only Peru measured agreement with physical child punishment, which was a significant risk factor for physical child punishment (only) and co-occurrence (supporting previous research on violent discipline [29, 36, 59, 61]), but not IPV (only). Support for wife-beating in Colombia and Peru was not significantly correlated with any pattern of violence except co-occurrence in Peru. This contrasts with other research [31, 35], including a previous analysis of the same Colombia dataset that found a significant association between support for wife-beating and beating children under five with objects [55]. This study replicated that finding by replacing ‘any physical child punishment’ with ‘beating with objects’, highlighting how differences in operational definitions may change findings, and comparisons should be made with caution. It is also noteworthy that support for wife-beating in these surveys was extremely low: only 1.4% of women in Peru and 3.4% of women in Colombia agreed with any reason for wife-beating, compared with 41.1% of women across 49 LMICs in a 2018 analysis [102]. Moreover, it is possible that community or male support for wife-beating may elevate the risk of violence even if female support does not, but that was beyond the scope of this study. The strongest evidence for shared risk factors was for caregiver histories of violence in childhood, providing support for ‘intergenerational transmission of violence’ theories [23, 67, 103]. Histories of almost all patterns of violence (caregiver violence, exposure to IPV and/or both) in childhood among women (all countries) and their partners (Colombia and Mexico) were significant correlates of all patterns of violence in the current household, with a couple of exceptions in Colombia.

Finally, this study found that associations between physical child punishment and IPV could not be explained by shared risk factors alone. In all countries, physical IPV (past year and before past year) remained a significant correlate for physical child punishment, even after controlling for all other factors. Understanding pathways by which IPV increases the risk of physical child punishment was beyond the scope of this study; however, these findings are consistent with the theory that partner violence may “spill over” into caregiver-child relationships due to stress, social learning and/or normalization of violence [21, 27, 46, 49].

### Limitations

This study had many limitations. Survivor reports of IPV and caregiver reports of violent discipline may underestimate prevalence due to social stigma or fear of reprisal. In contrast to the MICS [28], these surveys measured a small number of disciplinary acts in diverse ways, without asking about a specific child or timeframe. Nonetheless, the estimate of physical child punishment in Mexico produced by this analysis (39.6%, 95% CI: 38.9–40.4) was only somewhat lower than the Mexico 2015 MICS estimate of 43.7% [104], even though MICS asked about eight acts of physical punishment and ENDIREH asked about only one.

Another limitation was that while key sociodemographic variables (e.g. age, education, wealth, etc.) were highly comparable across surveys, other variables were not. As a result, models varied by country, and comparisons should be made with caution. In addition, this analysis was limited to physical violence, in contrast to studies that define child maltreatment and IPV to include sexual and emotional as well as physical abuse [21].

Another important limitation is that the ecological framework posits that societal, community, household and individual factors may contribute to violence [82, 83], but this analysis was limited to select household and individual variables based on data availability and a quest for comparability. Other correlates worthy of research include levels of armed/criminal violence and socio-economic disadvantages in the community [42, 54–58], inequitable gender norms endorsed by male partners [23, 31, 62], maternal anxiety, depression and harmful use of alcohol/drugs [26, 42, 48, 105, 106], and histories of unplanned or unwanted pregnancies [106]. Finally, an analysis of how correlates varied according to whether the woman, her partner or both physically punished the children was beyond the scope of this study but that is a worthy question for future research.

## Conclusions

This study highlights important intersections between violent discipline of children and IPV against women, consistent with calls for greater investment in coordinated responses to both forms of violence [107]. The study findings are consistent with several theories of change articulated by key international violence prevention frameworks [82, 83], including the theory that physical child punishment and IPV against women may share risk and protective factors and that more equitable gender relations, empowerment of women and collaborative partnerships may protect women and children from violence. These theories suggest a need for strategies to improve women’s access to education, to prevent child marriage/early motherhood and to promote more collaborative, gender equitable partnerships. This study also produced findings consistent with the theory that violence between partners may ‘spill over’ into caregiver-child relationships, regardless of shared risk factors, suggesting that to be effective, programmes aiming to reduce violent discipline of children may also need to address IPV against women in the household. Finally, evidence of intergenerational transmission suggests that long run prevention of violence against women may not be possible without reducing violent discipline of children, and vice versa. Generally, this study highlights a need for research, programmes and policies to pay greater attention to intersections between violence against children and violence against women.

## Supplementary Information

**Additional file 1:** **Supplemental Table A.** Missing responses.

**Additional file 2:** **Supplemental Table B.** Adjusted odds ratios of each pattern of physical violence, reduced models.

**Additional file 3:** **Supplemental Table C.** Crude and adjusted odds ratios of co-occurrence (physical child punishment and physical IPV).

## Data Availability

Datasets analysed in this study are open access and available online. The 2015 Colombia DHS dataset is available from DHS [https://dhsprogram.com/methodology/survey/survey-display-476.cfm] [89], The 2016 Mexico ENDIREH dataset from INEGI [https://www.inegi.org.mx/programas/endireh/2016/] [90] and the 2018 Peru DHS dataset from INEI [http://iinei.inei.gob.pe/microdatos/] [91].

## Abbreviations

aOR: Adjusted odds ratio
CI: Confidence interval
DHS: Demographic and Health Survey
DK: Don’t know
ENDIREH: Encuesta Nacional Sobre La Dinámica De Las Relaciones en Los Hogares
INEGI: Instituto Nacional de Estadística y Geografía
IPV: Intimate partner violence
LAC: Latin America and the Caribbean
LMIC: Low- and middle-income countries
MICS: Multiple Indicator Cluster Survey
NM: Not measured
OR: Odds ratio
PSU: Primary sampling unit
Ref: Reference category
SDG: Sustainable Development Goal
UN: United Nations
VIF: Variance inflation factor
