# Supplementary material for: Correlates of co-occurring physical child punishment and physical intimate partner violence in Colombia, Mexico and Peru
Source: BMC Public Health. 2022 Nov 28;22:2195. doi: 10.1186/s12889-022-14453-6 (PMC9702951; doi:10.1186/s12889-022-14453-6)
Supplement: Supplementary file 1 — Additional file 1: Supplemental Table A. Missing responses. [file 12889_2022_14453_MOESM1_ESM.docx]

**Supplemental Table A.** Missing responses*

|  | **Colombia** | **Mexico** | **Peru** |
| --- | --- | --- | --- |
| **Violence variables** |  |  |  |
| Physical child punishment | 0 | 0 | † |
| Physical IPV past year | 0 | 1 | 0 |
| Physical IPV ever | 0 | 0 | 0 |
| **Other variables** |  |  |  |
| Age | § | § | § |
| Education | 0 | 1 | 0 |
| Household wealth | 0 | 0 | 0 |
| Residence (e.g., urban, rural) | 0 | 0 | 0 |
| Indigenous ethnicity | 0 | 1 | 0 |
| Age of first marriage and/or birth <18 | 0 | 183 | 0 |
| Number of children aged 1-14 | 0 | 0 | 0 |
| Age of youngest child aged 1-14 | 0 | 0 | 0 |
| Husband/partner tries to socially isolate her | 0 | 0 | 0 |
| Husband/partner drinks to excess | NM | 0 | 0 |
| Joint decisions about money | 0 | 0 | 0 |
| She and her husband/partner share household chores | 0 | 4 | NM |
| She agrees wife beating justified for 1+ reason | 0 | NM | 0 |
| She believes physical punishment is necessary | NM | NM | 0 |
| Respondent exposed to IPV in childhood | 0 | 0 | 0 |
| Caregiver violence against respondent in childhood | 0 | 0 | 0 |
| Husband/partner exposed to IPV in childhood | 0 | 0 | NM |
| Caregiver violence against husband/partner in childhood | 0 | 0 | NM |

NM: Not measured.

* Violence and other composite variables were marked as missing if respondent was missing a response to at least one subitem and did not have a ‘yes’ to any other subitem in that composite variable.

† The Peru dataset did not distinguish between women who reported that ‘no one' punished the children versus women who did not respond at all to the filter question, "who punishes the children?". Together, these women comprised 6.3% of the study subsample, virtually the same percentage (6.5%) who reported 'no one' punishes the children in response to the same filter question in Colombia.

§ All surveys excluded women missing age.
