# Supplementary material for: Correlates of co-occurring physical child punishment and physical intimate partner violence in Colombia, Mexico and Peru
Source: BMC Public Health. 2022 Nov 28;22:2195. doi: 10.1186/s12889-022-14453-6 (PMC9702951; doi:10.1186/s12889-022-14453-6)
Supplement: Supplementary file 2 — Additional file 2: Supplemental Table B. Adjusted odds ratios of each pattern of physical violence, reduced models. [file 12889_2022_14453_MOESM2_ESM.docx]

**Supplemental Table B** Adjusted odds ratios of each pattern of physical violence, reduced model

| **COLOMBIA** | **Physical punishment only** | | | **Co-occurrence** | | | **Physical IPV only** | | |
| --- | --- | --- | --- | --- | --- | --- | --- | --- | --- |
| **Women's characteristics** | **aOR** | **CI (95%)** | ***p*-value** | **aOR** | **CI (95%)** | ***p*-value** | **aOR** | **CI (95%)** | ***p*-value** |
| **Age** (Ref: 40-49 years) |  |  |  |  |  |  |  |  |  |
| 15-29 years | 1.05 | 0.86-1.28 | 0.648 | **0.68** | **0.51**-**0.90** | **0.007** | 1.04 | 0.76-1.41 | 0.819 |
| 30-39 years | 1.06 | 0.86-1.31 | 0.580 | 0.80 | 0.64-1.01 | 0.060 | 1.20 | 0.91-1.59 | 0.195 |
| **Education** (Ref: Post-secondary) |  |  |  |  |  |  |  |  |  |
| Primary or none | 0.94 | 0.75-1.19 | 0.613 | **1.42** | **1.05**-**1.92** | **0.024** | 0.95 | 0.69-1.31 | 0.750 |
| Lower secondary | 0.95 | 0.74-1.22 | 0.700 | **1.61** | **1.18**-**2.19** | **0.003** | 1.33 | 0.92-1.93 | 0.128 |
| Upper secondary | 0.97 | 0.77-1.21 | 0.767 | **1.32** | **1.01**-**1.73** | **0.041** | 1.18 | 0.84-1.65 | 0.334 |
| **Household wealth** (Ref: Richest) |  |  |  |  |  |  |  |  |  |
| Poorest | 1.02 | 0.81-1.30 | 0.854 | **1.54** | **1.08**-**2.18** | **0.016** | 1.40 | 0.99-1.96 | 0.055 |
| Middle | 1.04 | 0.84-1.29 | 0.693 | 1.19 | 0.86-1.65 | 0.300 | 1.11 | 0.82-1.51 | 0.490 |
| **Urban residence** (Ref: Rural) | 0.88 | 0.74-1.05 | 0.158 | **1.28** | **1.02**-**1.61** | **0.032** | 1.14 | 0.88-1.46 | 0.319 |
| **Indigenous ethnicity** (Ref: No) | **0.66** | **0.54**-**0.81** | **<0.001** | **0.73** | **0.55**-**0.98** | **0.035** | 1.05 | 0.80-1.37 | 0.740 |
| **Married/mother age<18** (Ref:18+) | 1.02 | 0.86-1.22 | 0.784 | **1.28** | **1.03**-**1.60** | **0.025** | **1.49** | **1.18-1.89** | **0.001** |
| **2+ children aged 1-14** (Ref: 1) | **1.36** | **1.13**-**1.64** | **0.001** | **1.44** | **1.18**-**1.75** | **<0.001** | 0.93 | 0.75-1.16 | 0.518 |
| **Age of youngest child** (Ref: 1) |  |  |  |  |  |  |  |  |  |
| 2-5 years | **1.50** | **1.16**-**1.95** | **0.002** | **1.38** | **1.04**-**1.84** | **0.025** | 1.29 | 0.94-1.76 | 0.109 |
| 6-9 years | **1.34** | **1.04**-**1.75** | **0.027** | 1.12 | 0.82-1.55 | 0.475 | **1.48** | **1.07-2.07** | **0.019** |
| 10-14 years | 0.96 | 0.69-1.34 | 0.820 | 0.89 | 0.61-1.29 | 0.530 | **1.53** | **1.03-2.28** | **0.037** |
| **Partner isolates her** (Ref: No) | 1.00 | 0.79-1.25 | 0.969 | **6.75** | **5.37**-**8.49** | **<0.001** | **5.80** | **4.73-7.11** | **<0.001** |
| **Joint money decisions** (Ref: No) | **0.81** | **0.69**-**0.94** | **0.007** | **0.72** | **0.61**-**0.85** | **<0.001** | **0.61** | **0.50-0.74** | **<0.001** |
| **Violence in her childhood** (Ref: No) |  |  |  |  |  |  |  |  |  |
| Caregiver violence only | **2.94** | **2.43**-**3.56** | **<0.001** | **3.03** | **2.35**-**3.91** | **<0.001** | **1.30** | **1.03-1.64** | **0.029** |
| Co-occurrence | **3.17** | **2.58**-**3.90** | **<0.001** | **6.08** | **4.66**-**7.91** | **<0.001** | **1.74** | **1.35-2.23** | **<0.001** |
| Exposure to IPV only | 1.18 | 0.86-1.61 | 0.314 | **2.12** | **1.45**-**3.10** | **<0.001** | **1.61** | **1.15-2.23** | **0.005** |
| **MEXICO** | **Physical punishment only** | | | **Co-occurrence** | | | **Physical IPV only** | | |
| **Women's characteristics** | **aOR** | **CI (95%)** | ***p*-value** | **aOR** | **CI (95%)** | ***p*-value** | **aOR** | **CI (95%)** | ***p*-value** |
| **Age** (Ref: 40-49 years) |  |  |  |  |  |  |  |  |  |
| 15-29 years | **0.73** | **0.65**-**0.82** | **<0.001** | 0.83 | 0.69-1.01 | 0.059 | 0.92 | 0.73-1.16 | 0.488 |
| 30-39 years | **0.90** | **0.81**-**0.99** | **0.026** | 1.05 | 0.90-1.21 | 0.554 | 1.02 | 0.86-1.22 | 0.787 |
| **Education** (Ref: Post-secondary) |  |  |  |  |  |  |  |  |  |
| Primary or none | 1.01 | 0.89-1.16 | 0.825 | **1.52** | **1.21**-**1.91** | **<0.001** | **1.52** | **1.18-1.96** | **0.001** |
| Lower secondary | **1.18** | **1.05**-**1.32** | **0.005** | **1.60** | **1.31**-**1.96** | **<0.001** | **1.66** | **1.31-2.12** | **<0.001** |
| Upper secondary | **1.15** | **1.02**-**1.29** | **0.020** | **1.59** | **1.30**-**1.94** | **<0.001** | **1.31** | **1.02-1.70** | **0.035** |
| **Household wealth** (Ref: Richest) |  |  |  |  |  |  |  |  |  |
| Poorest | 1.07 | 0.96-1.20 | 0.243 | **1.37** | **1.14**-**1.64** | **0.001** | **1.33** | **1.08-1.64** | **0.008** |
| Middle | 1.08 | 0.98-1.19 | 0.118 | **1.25** | **1.05**-**1.48** | **0.011** | 1.17 | 0.96-1.41 | 0.116 |
| **Urban residence** (Ref: Rural) | **0.84** | **0.78**-**0.92** | **<0.001** | 1.09 | 0.96-1.24 | 0.181 | **1.26** | **1.09-1.45** | **0.002** |
| **Indigenous ethnicity** (Ref: No) | **0.71** | **0.61**-**0.81** | **<0.001** | 0.99 | 0.81-1.22 | 0.935 | 1.10 | 0.90-1.34 | 0.363 |
| **Married/mother age<18** (Ref:18+) | 0.94 | 0.86-1.02 | 0.131 | **1.38** | **1.22**-**1.57** | **<0.001** | **1.29** | **1.11-1.51** | **0.001** |
| **2+ children aged 1-14** (Ref: 1) | **1.69** | **1.56**-**1.82** | **<0.001** | **1.71** | **1.52**-**1.92** | **<0.001** | **1.19** | **1.03-1.38** | **0.018** |
| **Age of youngest child** (Ref: 1) |  |  |  |  |  |  |  |  |  |
| 2-5 years | **1.49** | **1.31**-**1.69** | **<0.001** | **1.53** | **1.27**-**1.84** | **<0.001** | 1.10 | 0.89-1.36 | 0.381 |
| 6-9 years | **1.46** | **1.28**-**1.68** | **<0.001** | **1.62** | **1.31**-**2.00** | **<0.001** | 1.11 | 0.88-1.40 | 0.385 |
| 10-14 years | **1.18** | **1.01**-**1.37** | **0.039** | **1.49** | **1.18**-**1.89** | **0.001** | 1.25 | 0.96-1.62 | 0.096 |
| **Partner isolates her** (Ref: No) | **1.99** | **1.73**-**2.30** | **<0.001** | **9.47** | **8.10**-**11.07** | **<0.001** | **7.35** | **6.18-8.74** | **<0.001** |
| **Joint money decisions** (Ref: No) | **0.90** | **0.84**-**0.97** | **0.005** | **0.60** | **0.54**-**0.67** | **<0.001** | **0.67** | **0.58-0.76** | **<0.001** |
| **Violence in her childhood** (Ref: No) |  |  |  |  |  |  |  |  |  |
| Caregiver violence only | **4.20** | **3.84**-**4.61** | **<0.001** | **5.53** | **4.78**-**6.41** | **<0.001** | **2.33** | **1.93-2.81** | **<0.001** |
| Co-occurrence | **3.40** | **3.05**-**3.79** | **<0.001** | **8.92** | **7.70**-**10.34** | **<0.001** | **3.60** | **3.02-4.28** | **<0.001** |
| Exposure to IPV only | **1.96** | **1.75**-**2.20** | **<0.001** | **3.27** | **2.74**-**3.91** | **<0.001** | **2.56** | **2.11-3.10** | **<0.001** |

**Supplemental Table B** Adjusted odds ratios of each pattern of physical violence, reduced model (cont.)

| **PERU** | **Physical punishment only** | | | **Co-occurrence** | | | **Physical IPV only** | | |
| --- | --- | --- | --- | --- | --- | --- | --- | --- | --- |
| **Women's characteristics** | **aOR** | **CI (95%)** | ***p*-value** | **aOR** | **CI (95%)** | ***p*-value** | **aOR** | **CI (95%)** | ***p*-value** |
| **Age** (Ref: 40-49 years) |  |  |  |  |  |  |  |  |  |
| 15-29 years | **1.33** | **1.06**-**1.65** | **0.013** | 1.13 | 0.83-1.53 | 0.429 | **0.70** | **0.51**-**0.95** | **0.022** |
| 30-39 years | 1.06 | 0.86-1.29 | 0.597 | 1.06 | 0.81-1.38 | 0.675 | 0.91 | 0.70-1.17 | 0.455 |
| **Education** (Ref: Post-secondary) |  |  |  |  |  |  |  |  |  |
| Primary or none | **1.64** | **1.28**-**2.12** | **<0.001** | 1.25 | 0.91-1.72 | 0.174 | 1.03 | 0.71-1.49 | 0.870 |
| Lower secondary | 1.25 | 0.95-1.65 | 0.109 | 1.10 | 0.79-1.54 | 0.559 | 1.13 | 0.77-1.64 | 0.530 |
| Upper secondary | 1.16 | 0.96-1.41 | 0.124 | 1.04 | 0.80-1.36 | 0.745 | 1.05 | 0.79-1.40 | 0.727 |
| **Household wealth** (Ref: Richest) |  |  |  |  |  |  |  |  |  |
| Poorest | 1.03 | 0.80-1.33 | 0.795 | **1.39** | **1.01**-**1.90** | **0.041** | **1.59** | **1.13**-**2.25** | **0.008** |
| Middle | 0.96 | 0.78-1.19 | 0.708 | 1.06 | 0.79-1.43 | 0.694 | 1.33 | 0.98-1.80 | 0.068 |
| **Urban residence** (Ref: Rural) | 1.03 | 0.86-1.23 | 0.740 | **1.28** | **1.02**-**1.62** | **0.036** | **1.62** | **1.27**-**2.07** | **<0.001** |
| **Indigenous ethnicity** (Ref: No) | **0.66** | **0.55**-**0.79** | **<0.001** | 1.06 | 0.85-1.32 | 0.619 | **1.42** | **1.13**-**1.78** | **0.002** |
| **Married/mother age<18** (Ref:18+) | 1.01 | 0.86-1.18 | 0.926 | 1.21 | 0.99-1.49 | 0.069 | **1.37** | **1.07**-**1.76** | **0.013** |
| **2+ children aged 1-14** (Ref: 1) | **1.64** | **1.41**-**1.91** | **<0.001** | **1.86** | **1.48**-**2.34** | **<0.001** | 1.22 | 0.98-1.51 | 0.070 |
| **Age of youngest child** (Ref: 1) |  |  |  |  |  |  |  |  |  |
| 2-5 years | **1.78** | **1.56**-**2.03** | **<0.001** | **1.75** | **1.45-2.10** | **<0.001** | **1.35** | **1.10**-**1.65** | **0.004** |
| 6-9 years | **1.30** | **1.06**-**1.59** | **0.013** | **1.47** | **1.13**-**1.92** | **0.005** | **1.44** | **1.09**-**1.91** | **0.010** |
| 10-14 years | 0.80 | 0.57-1.10 | 0.169 | 1.32 | 0.84-2.09 | 0.234 | **1.47** | **1.02**-**2.12** | **0.039** |
| **Partner isolates her** (Ref: No) | **1.46** | **1.06**-**2.01** | **0.021** | **6.35** | **4.86**-**8.29** | **<0.001** | **6.40** | **4.80**-**8.55** | **<0.001** |
| **Joint money decisions** (Ref: No) | 0.89 | 0.76-1.04 | 0.152 | **0.71** | **0.57**-**0.90** | **0.004** | **0.76** | **0.61**-**0.94** | **0.013** |
| **Violence in her childhood** (Ref: No) |  |  |  |  |  |  |  |  |  |
| Caregiver violence only | **2.95** | **2.45**-**3.54** | **<0.001** | **3.19** | **2.25**-**4.51** | **<0.001** | **1.50** | **1.17**-**1.93** | **0.002** |
| Co-occurrence | **3.28** | **2.70**-**3.97** | **<0.001** | **5.89** | **4.20**-**8.25** | **<0.001** | **3.13** | **2.39**-**4.10** | **<0.001** |
| Exposure to IPV only | **1.64** | **1.22**-**2.21** | **0.001** | **2.64** | **1.68**-**4.13** | **<0.001** | **1.71** | **1.15**-**2.54** | **0.008** |

aOR: adjusted odds ratios; CI: Confidence interval; DK: doesn't know; IPV: intimate partner violence; Ref: reference category; significant *p*-values are bolded.
