# Supplementary material for: Correlates of co-occurring physical child punishment and physical intimate partner violence in Colombia, Mexico and Peru
Source: BMC Public Health. 2022 Nov 28;22:2195. doi: 10.1186/s12889-022-14453-6 (PMC9702951; doi:10.1186/s12889-022-14453-6)
Supplement: Supplementary file 3 — Additional file 3: Supplemental Table C. Crude and adjusted odds ratios of co-occurrence (physical child punishment and physical IPV). [file 12889_2022_14453_MOESM3_ESM.docx]

**Supplemental Table C** Crude and adjusted odds ratios of co-occurrence (physical child punishment and physical IPV)

|  | **COLOMBIA** | | | | | | **MEXICO** | | | | | | | **PERU** | | | | | | | | | |
| --- | --- | --- | --- | --- | --- | --- | --- | --- | --- | --- | --- | --- | --- | --- | --- | --- | --- | --- | --- | --- | --- | --- | --- |
|  | Crude | | Reduced model† | | Full model†† | | Crude | | Reduced model† | | Full model†† | | Crude | | | Reduced model† | | | | | Full model†† | | |
| **Women's characteristics** | **OR** | | **aOR** | | **aOR** | | **OR** | | **aOR** | | **aOR** | | **OR** | | | **aOR** | |  | | **aOR** | |  | |
| **Age** (Ref: 40-49 years) |  |  |  |  |  |  |  |  |  |  |  |  |  | |  | |  | |  | |  | |  |
| 15-29 years | 0.99 |  | **0.68** | ** | **0.69** | * | 0.89 |  | 0.83 |  | 0.84 |  | 1.14 | |  | | 1.13 | |  | | 1.11 | |  |
| 30-39 years | 0.97 |  | 0.80 |  | 0.79 |  | **1.14** | * | 1.05 |  | 1.08 |  | 1.18 | |  | | 1.06 | |  | | 1.06 | |  |
| **Education** (Ref: Post-secondary) |  |  |  |  |  |  |  |  |  |  |  |  |  | |  | |  | |  | |  | |  |
| Primary or none | **1.99** | *** | **1.42** | * | 1.35 |  | **2.54** | *** | **1.52** | *** | **1.35** | * | **1.85** | | *** | | 1.25 | |  | | **1.44** | | * |
| Lower secondary | **2.38** | *** | **1.61** | ** | **1.52** | * | **2.30** | *** | **1.60** | *** | **1.39** | ** | **1.88** | | *** | | 1.10 | |  | | 1.27 | |  |
| Upper secondary | **1.76** | *** | **1.32** | * | 1.29 |  | **1.78** | *** | **1.59** | *** | **1.38** | ** | **1.47** | | ** | | 1.04 | |  | | 1.14 | |  |
| **Household wealth** (Ref: Richest) |  |  |  |  |  |  |  |  |  |  |  |  |  | |  | |  | |  | |  | |  |
| Poorest | **1.63** | *** | **1.54** | * | **1.54** | ***** | **1.85** | *** | **1.37** | ** | 1.14 |  | **1.72** | | *** | | **1.39** | | * | | 1.30 | |  |
| Middle | **1.48** | * | 1.19 |  | 1.20 |  | **1.55** | *** | **1.25** | * | 1.12 |  | **1.39** | | * | | 1.06 | |  | | 1.01 | |  |
| **Residence** (Ref: Rural) |  |  |  |  |  |  |  |  |  |  |  |  |  | |  | |  | |  | |  | |  |
| Urban§ | 0.93 |  | **1.28** | * | **1.31** | ***** | 0.96 |  | 1.09 |  | **1.22** | * | 0.86 | |  | | **1.28** | | * | | **1.41** | | ** |
| Semi-urban | NM |  |  |  |  |  | 1.06 |  | § |  | 1.15 |  | NM | |  | |  | |  | | NM | |  |
| **Indigenous ethnicity** (Ref: Not) | 0.82 |  | **0.73** | * | **0.74** | ***** | 1.15 |  | 0.99 |  | 1.03 |  | **1.30** | | ** | | 1.06 | |  | | 0.96 | |  |
| **Married/mother age <18** (Ref:18+) | **1.60** | *** | **1.28** | * | **1.28** | ***** | **1.78** | *** | **1.38** | *** | **1.34** | *** | **1.54** | | *** | | 1.21 | |  | | 1.18 | |  |
| **2+ children aged 1-14** (Ref: 1) | **1.66** | *** | **1.44** | *** | **1.51** | *** | **1.73** | *** | **1.71** | *** | **1.70** | *** | **2.06** | | *** | | **1.86** | | *** | | **1.73** | | *** |
| **Age of youngest child** (Ref: 1) |  |  |  |  |  |  |  |  |  |  |  |  |  | |  | |  | |  | |  | |  |
| 2-5 years | **1.42** | * | **1.38** | * | **1.39** | * | **1.60** | *** | **1.53** | *** | **1.62** | *** | **1.72** | | *** | | **1.75** | | *** | | **1.74** | | *** |
| 6-9 years | 1.35 |  | 1.12 |  | 1.14 |  | **1.81** | *** | **1.62** | *** | **1.68** | *** | **1.40** | | ** | | **1.47** | | ** | | **1.42** | | * |
| 10-14 years | 0.93 |  | 0.89 |  | 0.92 |  | **1.57** | *** | **1.49** | ** | **1.56** | *** | 1.01 | |  | | 1.32 | |  | | 1.20 | |  |
| **Partner isolates her** (Ref: No) | **7.55** | *** | **6.75** | *** | **5.72** | *** | **11.99** | *** | **9.47** | *** | **6.76** | *** | **7.29** | | *** | | **6.35** | | *** | | **6.00** | | *** |
| **Partner drinks to excess** (Ref: No) | NM |  |  |  |  |  | **5.52** | *** |  |  | **3.35** | *** | **3.01** | | *** | |  | |  | | **2.47** | | *** |
| **Joint money decisions** (Ref: No) | **0.70** | *** | **0.72** | *** | **0.77** | ** | **0.51** | *** | **0.60** | *** | **0.67** | *** | **0.64** | | *** | | **0.71** | | ** | | **0.74** | | * |
| **Shared chores** (Ref: No) | **0.55** | *** |  |  | **0.70** | ** | **0.59** | *** |  |  | **0.74** | *** | NM | |  | |  | |  | | NM | |  |
| **Wife beating justified** (Ref: No/DK) | **1.40** | ** |  |  | 1.40 |  | NM |  |  |  | NM |  | **2.13** | | ** | |  | |  | | **1.75** | | * |
| **Phys. pun. necessary** (Ref: No/DK) | NM |  |  |  |  |  | NM |  |  |  | NM |  | **5.11** | | *** | |  | |  | | **4.50** | | *** |
| **Violence in her childhood** (Ref: None) | |  |  |  |  |  |  |  |  |  |  |  | |  |  | |  | |  | |  | |  |
| Caregiver violence only | **2.77** | *** | **3.03** | *** | **2.91** | *** | **5.74** | *** | **5.53** | *** | **4.33** | *** | **3.17** | | *** | | **3.19** | | *** | | **2.67** | | *** |
| Co-occurrence | **6.14** | *** | **6.08** | *** | **5.20** | *** | **11.23** | *** | **8.92** | *** | **6.28** | *** | **5.88** | | *** | | **5.89** | | *** | | **5.12** | | *** |
| Exposure to IPV only | **2.25** | *** | **2.12** | *** | **1.83** | ** | **3.90** | *** | **3.27** | *** | **2.46** | *** | **2.54** | | *** | | **2.64** | | *** | | **2.62** | | *** |
| **Violence in partner's childhood** (Ref: None/DK) | | | |  |  |  |  |  |  |  |  |  | |  |  | |  | |  | |  | |  |
| Caregiver violence only | **2.57** | *** |  |  | **2.14** | *** | **4.27** | *** |  |  | **2.45** | *** | NM | |  | |  | |  | | NM | |  |
| Co-occurrence | **5.35** | *** |  |  | **3.98** | *** | **7.84** | *** |  |  | **3.62** | *** | NM | |  | |  | |  | | NM | |  |
| Exposure to IPV only | **2.98** | *** |  |  | **2.49** | *** | **4.69** | *** |  |  | **2.67** | *** | NM | |  | |  | |  | | NM | |  |

aOR: adjusted odds ratio; DK: don't know; NM: mot measured; Phys.: physical; Ref: reference category; *p-*value* < 0.05; **p-*value* < 0.01; ***p-*value* < 0.001.
† Reduced model: Adjusted for only those variables measured by all three surveys.
†† Full models: Adjusted for all available variables in each country.
§ In Mexico, urban and semi-urban were combined (as ‘urban’) in the reduced model but separated in the full model.
